# Green synthesis of highly functionalized heterocyclic bearing pyrazole moiety for cancer-targeted chemo/radioisotope therapy

**DOI:** 10.1186/s13065-023-01053-7

**Published:** 2023-10-18

**Authors:** Kurls E. Anwer, Galal H. Sayed, Basma M. Essa, Adli A. Selim

**Affiliations:** 1 Heterocyclic Synthesis Lab, Chemistry Department, Faculty of Science, Ain Shams University, Abbassia, Cairo, 11566 Egypt; 2 Radioactive Isotopes and Generators Department, Egyptian Atomic Energy Authority, Cairo, 13759 Egypt; 3 Labelled Compounds Department, Egyptian Atomic Energy Authority, Cairo, 13759 Egypt

**Keywords:** Microwave, Grinding, Pyrazole, Radioiodination, Dual cancer therapy

## Abstract

New derivatives of heterocyclic bearing pyrazole moiety were synthesized (eight new compounds from 2 to 9) via green synthesis methods (microwave-assisted and grinding techniques). 4,6-Diamino-1,3-diphenyl-1*H*-pyrazolo[3,4-*b*]pyridine-5-carbonitrile (2) shows high anti-cancer activity against both HepG2 and HCT-116 with IC_50_ of 9.2 ± 2.8 and 7.7 ± 1.8 µM, respectively, which referenced to 5-Fu which is showing activity of 7.86 ± 0.5 and 5.35 ± 0.3 against both HepG2 and HCT-116, respectively. The cytotoxic activity against HCT-116 and HepG2 was slightly decreased and slightly increased, respectively, by a different pyrazole moiety (compound 5). Pharmacokinetics of compound 2 was carried out using the radioiodination technique in tumour-bearing Albino mice which shows good uptake at the tumour site. The biodistribution showed high accumulation in tumour tissues with a ratio of 13.7% ID/g organ after one hour in comparison with 2.97% ID/g organ at normal muscle at the same time point. As I-131 has maximum beta and gamma energies of 606.3 and 364.5 keV, respectively, therefore the newly synthesized compound 2 may be used for chemotherapy and TRT.

**Supplementary Information:**

The online version contains supplementary material available at 10.1186/s13065-023-01053-7.

## Introduction

Heterocyclic chemistry is a significant field of organic chemistry and has received a great deal of interest for its potential applications in industry, biology, and the advancement of human society [1–3]. Heterocyclic compounds typically have at least one heteroatom in their cyclic structures, such as nitrogen, sulfur, or oxygen can be made both naturally and artificially [3–5]. Molecules which provided by a heterocyclic nucleus mainly have improvements in salt formation properties and solubility which make an improvement in pharmacokinetic parameters [6, 7]. Additionally, it makes it easier to settle the pharmacophores and create several biologically active compounds such as anti-inflammatory, anti-tumor, anti-viral and anti-bacterial compounds [8–13]. Compounds containing pyrazole moiety have received widely attention among heterocyclic compounds as a result of its extensive use in drug development research.

Pyrazole compounds are one of the most important heterocyclic derivatives widely used in petrochemical industry [14], catalytic and polymer manufacturing [15, 16]. Pyrazole derivatives have partially harmful effects on environment and humans and there are requirements for converting them into safe and useful products [17, 18]. During the last decades, derivatives of pyrazole showed wide-range of biological and pharmacological activities such as: anti-inflammatory, antagonist, analgesic, anthelmintic, anticancer, herbicidal, antiviral, antimitotic, antioxidant, insecticidal and antimicrobial activities [19–26]. Furthermore, synthetic heterocyclic compounds containing nitrogen atoms have proven to have significant and diverse therapeutic potential for cancer which is one of the major causes of death worldwide [27].

Green chemistry is the chemical processes design science that eliminate or reduce the hazardous compounds generation. It prevents the pollution at a molecular level. Microwave and grinding techniques use in heterocyclic compounds synthesis are also important branch from green chemistry techniques. One-pot multicomponent reactions [28, 29] are one of the most important tools for synthesis with facile execution, the effectiveness for its productivity and highly diverse products generation in a single running and from easily starting materials. So, such techniques have much more attention because of its safe on the environment, improvement of the reactions yield and time, more convenient, and easily synthetic procedures which are highly energy efficient. Comparing both microwave irradiation and grinding techniques with the conventional heating method; it’s clear that microwave irradiation and grinding techniques are more environmentally tolerant, easily controlled and friendly environmental. As advantage, many heterocyclic reactions were carried out in shorter reaction time, higher yield, and milder and cleaner conditions [30–32]. So now, this green synthesis type is considered as significant technique in heterocyclic chemistry synthesis because of its economy, simplicity and mild conditions. as aforementioned about heterocycles derivatives, and according to their favorite applications in biology and industry; more efforts in synthesizing novel heterocycles derivatives still continued [33–40].

Nuclear medicine technology is essential for developing both the search for disease treatments and the development of novel drugs. Iodine-131 is one of the most used radioisotopes in nuclear medicine. I-131 decays to the extremely stable xenon-131 with a half-life of 8.03 days. A negative beta particle as well as gamma photons are released during the decay process. The major beta emission has average and maximum energies of 191.5 keV and 606.3 keV, respectively, while the primary photon (gamma emission) has an energy of 364.5 keV [41], therefore it used in targeted radionuclide therapies (TRTs) [42].

**Scheme 1:**
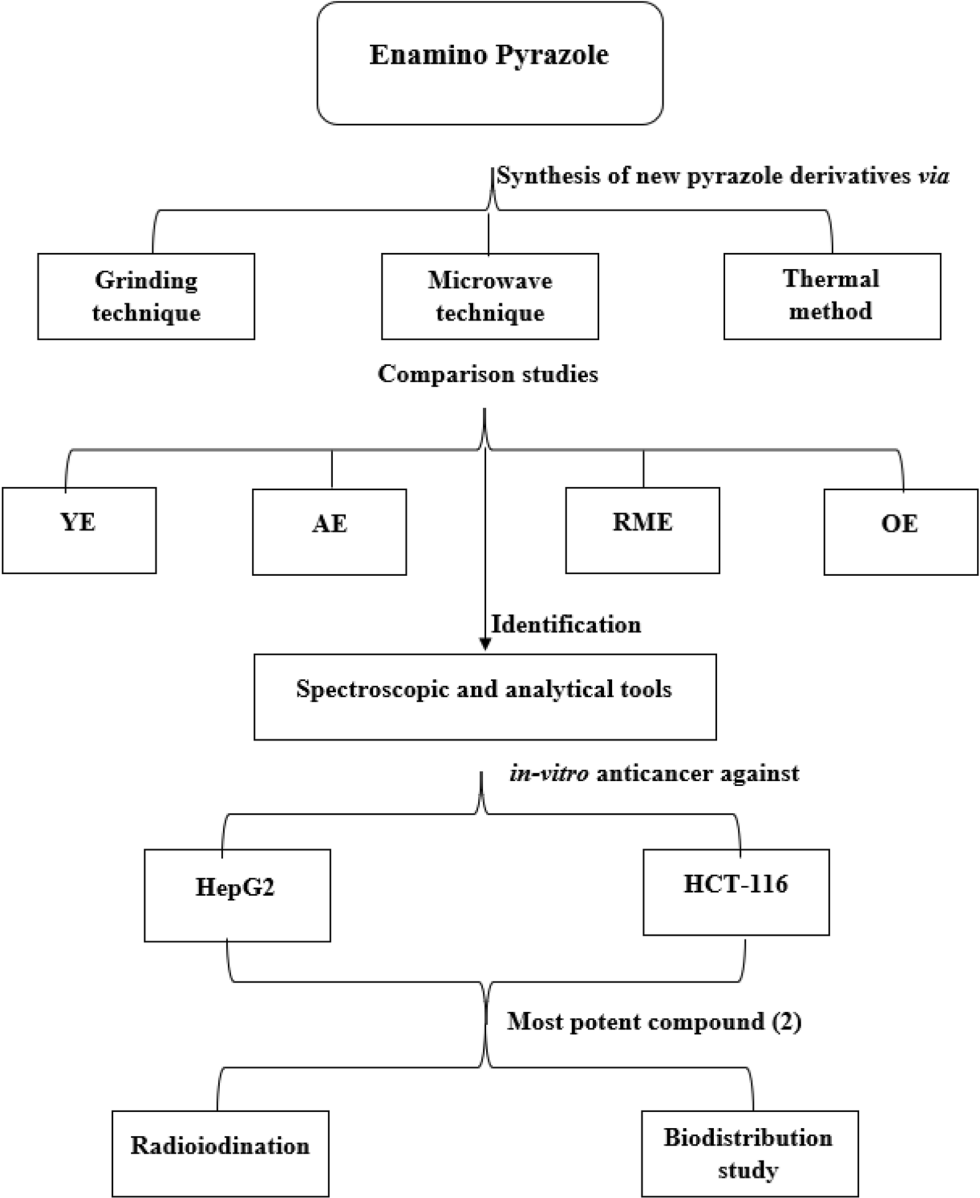
Working plan

In this study, designing and synthesizing enamino pyrazole derivative was reported as starting material via one-pot multicomponent reaction using microwave and grinding techniques. On the other hand, the reactions were pressed using thermal method according to literature method [43, 44]. Comparison between the percentage yields and consumed times, which resulting from the two techniques were performed. The yield economy “YE”, atom economy “AE”, reaction mass efficiency “RME” and optimum efficiency “OE” were used for comparison between the consumed times and the percent of the yields resulting from these techniques. The various synthesized compounds were illustrated by using different spectroscopic and analytical tools. The newly compounds were screened *in-vitro* anticancer against two cancer cell lines, namely, hepatocellular carcinoma (HepG2), and colorectal carcinoma (HCT-116). Radioiodination technique was used to study the pharmacokinetic of the most effective synthesized compound and also to be used as a potential agent as TRT for cancer tissues (Scheme 1).

## Experimental

### Synthesis

All solvents, reagents and chemicals were bought from Sigma Aldrich. TLC (Merck Kiesel gel 60F254, BDH) to monitor the progress of all synthesized compounds. Microwave reactions were carried out with microwave reactor Anton Paar (monowave 300). All melting points were measured on a digital Stuart electric melting point apparatus “SMP3”. Infrared spectra measurements were determined using KBr disks on PerkinElmer 293 spectrophotometer. The ^1^H-NMR and ^13^C-NMR spectra were measured on a Varian Mercury 75 MHz spectrometer. A GC-2010 Shimadzu Gas chromatography mass spectrometer (EI, 70 eV) was used for Mass spectrometry measurements. A Perkin-Elmer CHN-2400 analyzer was used for elemental microanalyses (CHN). No-carrier-added [^131^I] NaI was received as a gift from RPF (Radioisotopes-Production-Facility), Egyptian Atomic Energy Authority (EAEA). A NaI (Tl) scintillation counter (Scaler Ratemeter SR7 model, the United Kingdom) was used for γ-ray radioactivity measurement.

#### General procedure for preparation of compounds (2–4)

A mixture of enaminonitrile 1 (10 mmol., 2.6 g) and each of malononitrile (10 mmol., 0.66 g), cyanoacetamide (10 mmol., 0.84 g) and/or chloroacetic acid (10 mmol., 0.94 mL) in presence of sodium ethoxide (0.4 g sodium ethoxide in 2 mL ethanol) was irradiated for 2–2.5 min. The reaction mixture after cooling was poured into ice water, formed solid was filtrated, and then recrystallized from the proper solvent to give compounds 2–4, respectively.

##### 4,6-Diamino-1,3-diphenyl-1H-pyrazolo[3,4-b]pyridine-5-carbonitrile (2)

Black crystals from ethanol. Yield 96.7%. IR (cm^−1^) υ: 3442, 3315, 3290 (2NH_2_), 2169 (CN), 1633, 1601 (C = C). ^1^H-NMR (300 MHz, DMSO-*d*_6_) δ (ppm): 6.72–7.87 (m, 10H; Ar–H), 10.32 (s, 4H, 2NH_2_; D_2_O exchangeable). MS (m/z): 326 (M^+^, 40.9%). Anal. Calcd. for C_19_H_14_N_6_: C, 69.92; H, 4.32; N, 25.75; Found: C, 69.88; H, 4.14; N, 25.98%.

##### 4,6-Diamino-1,3-diphenyl-1H-pyrazolo[3,4-b]pyridine-5-carboxamide (3)

Orange crystals from ethanol. Yield 94.5%. IR (cm^−1^) υ: 3445, 3309, 3281 (NH_2_), 1658 (C = O), 1595, 1564 (C = N). ^1^H-NMR (300 MHz, DMSO-*d*_6_) δ (ppm): 6.72–7.86 (m, 10H; Ar–H), 8.89 (s, 4H, 2NH_2_; D_2_O exchangeable), 11.24 (s, 2H, CONH_2_; D_2_O-exchangeable); ^13^C-NMR (75 MHz, DMSO-*d*_6_) δ (ppm): 117.1, 123.9, 130.7, 133.0, 133.7, 134.2, 140.9, 141.6, 150.4 and 162.3. MS (m/z): 344 (M^+^, 13.3%). Anal. Calcd. for C_19_H_16_N_6_O: C, 66.27; H, 4.68; N, 24.40; Found: C, 66.05; H, 4.74; N, 24.51%.

##### 4-Amino-1,3-diphenyl-1,6-dihydropyrrolo[2,3-c]pyrazole-5-carboxylic acid (4)

Red crystals from ethanol. Yield 95.5%. IR (cm^−1^) υ: 3476 (OH), 3414, 3311 (NH_2_), 3223 (NH), 1709 (C = O), 1645(C = N), 1593(C = C). ^1^H-NMR (300 MHz, DMSO-*d*_6_) δ (ppm): 5.08 (s, 2H, NH_2_; D_2_O exchangeable), 6.72–7.89 (m, 10H; Ar–H), 10.0 (s, 1H, NH; D_2_O exchangeable), 10.40 (s, 1H, OH; D_2_O exchange). MS (m/z): 318 (M^+^, 9.8%). Anal. Calcd. for C_18_H_14_N_4_O_2_: C, 67.92; H, 4.43; N, 17.60; Found: C, 67.81; H, 4.44; N, 17.51%.

#### 4,6-Diphenyl-1,6-dihydropyrazolo[3,4-c]pyrazol-3-amine (5)

A mixture of enaminonitrile 1 (10 mmol., 2.6 g) and hydrazine hydrate (10 mmol., 0.5 mL) was irradiated for 4 min. The reaction mixture after cooling was poured into ice water, formed solid was filtrated, and recrystallized from methanol to give compound 5.

Brown crystals. Yield 94.1%. IR (cm^−1^) υ: 3385, 3313 (NH_2_), 3201 (NH), 1627, 1592 (C = N). ^1^H-NMR (300 MHz, DMSO-*d*_6_) δ (ppm): 6.73–7.87 (m, 10H; Ar–H), 8.68 (s, 1H, NH; D_2_O exchangeable), 10.31 (s, 2H, NH_2_; D_2_O exchangeable). MS (m/z): 275 (M^+^(8.1%). Anal. Calcd. for C_16_H_13_N_5_: C, 69.80; H, 4.76; N, 25.44; Found: C, 69.67; H, 4.88; N, 25.45%.

#### 4-Amino-1,3-diphenyl-1,7-dihydro-6H-pyrazolo[3,4-d]pyrimidin-6-one (6)

A mixture of enaminonitrile 1 (10 mmol., 2.6 g), urea (10 mmol., 0.66 g) and acetic acid (2 mL) was irradiated for 3 min. The reaction mixture after cooling was poured into ice water, formed solid was filtrated, and recrystallized from toluene to afford compound **6**.

Red crystals. Yield 93.3%. IR (cm^−1^) υ: 3456, 3312 (NH_2,_ NH), 1688 (C = O), 1611 (C = N). ^1^H-NMR (300 MHz, DMSO-*d*_6_) δ (ppm): 6.69–7.92 (m, 10H; Ar–H), 9.55 (s, 2H, NH_2_; D_2_O exchangeable), 10.28 (s, 1H, NH; D_2_O exchangeable); ^13^C-NMR (75 MHz, DMSO-*d*_6_) δ (ppm): 117.1, 123.3, 130.2, 133.6, 134.1, 134.2, 140.9, 141.6, 150.4 and 159.8. MS (m/z): 303 (M^+^, 37.7%). Anal. Calcd. for C_17_H_13_N_5_O: C, 67.32; H, 4.32; N, 23.09; Found: C, 67.14; H, 4.39; N, 23.44%.

#### General procedure for preparation of compounds (7 & 8)

A mixture of enaminonitrile 1 (10 mmol., 2.6), and each of thiourea (10 mmol., 0.76 g) and/ or benzaldehyde (10 mmol., 1.06 mL) in the presence of sodium ethoxide (0.4 g sodium ethoxide in 2 mL ethanol) was irradiated for 2.5 min. The reaction mixture after cooling was poured into ice water, formed solid was filtrated, and then recrystallized from the proper solvent to give compounds 7&8.

##### 4-Amino-1,3-diphenyl-1,7-dihydro-6H-pyrazolo[3,4-d]pyrimidine-6-thione (7)

Red crystals from toluene. Yield 96.6%. IR (cm^−1^) υ: 3397, 3311 (NH_2_), 3200 (NH), 1600, 1593 (C = N), 1287 (C = S). ^1^H-NMR (300 MHz, DMSO-*d*_6_) δ (ppm): 6.72–7.87 (m, 10H, Ar–H), 10.33 (s, 2H, NH_2_, D_2_O exchangeable), 13.14 (s, 1H, NH, D_2_O exchangeable). ^13^C-NMR (75 MHz, DMSO-*d*_6_) δ (ppm): 117.2, 123.9, 130.7, 133.0, 133.8, 134.1, 134.2, 140.9, 141.6, 150.5 and 189.1. MS (m/z): 319 (M^+^, 10.7%). Anal. Calcd. for C_17_H_13_N_5_S: C, 63.93; H, 4.10; N, 21.93; S, 10.04; Found: C, 63.74; H, 4.22; N, 22.04; S, 10.00%.

###### 4-Ethoxy-1,3,6-triphenyl-1H-pyrazolo[3,4-d]pyrimidine (8)

Brown crystals from ethanol. Yield 95.2%. IR (cm^−1^) υ: 1633, 1600 (C = N). ^1^H-NMR (300 MHz, DMSO-*d*_6_) δ (ppm): 1.31 (t, *J* = 6.0 Hz, 3H; CH_3_CH_2_), 4.20 (q, *J* = 6.6 Hz, 2H; CH_3_CH_2_), 6.72–7.86 (m, 15H; Ar–H); ^13^C-NMR (75 MHz, DMSO-*d*_6_) δ (ppm): 15.9, 56.8, 111.9, 118.8, 125.6, 127.9, 127.9, 135.7, 136.6 and 145.2. MS (m/z): 392 (M^+^, 14.1%). Anal. Calcd. for C_25_H_20_N_4_O: C, 76.51; H, 5.14; N, 14.28; Found: C, 76.43; H, 5.28; N, 14.01%.

#### 6-(5-Amino-1,3-diphenyl-1H-pyrazol-4-yl)-1,3-diphenyl-1H-pyrazolo[3,4-d]pyrimidin-4-amine (9)

A mixture of enaminonitrile 1 (10 mmol., 2.6 g) and TEA (2 mL) was irradiated for 3 min. The reaction mixture after cooling was poured into ice water, formed solid was filtrated, and recrystallized from methanol to afford compound 9.

Red crystals. Yield 97.4%. IR (cm^−1^) υ: 3427, 3311 (NH_2_), 1591 (C = N). ^1^H-NMR (300 MHz, DMSO-*d*_6_) δ (ppm): 6.72–7.86 (m, 20H; Ar–H), 9.41 (s, 2H, NH_2_; D_2_O exchangeable), 10.57 (s, 2H, NH_2_; D_2_O exchangeable); ^13^C-NMR (75 MHz, DMSO-*d*_6_) δ (ppm): 112.0, 118.7, 125.6, 127.9, 128.6, 129.1, 135.9, 136.4 and 145.3. MS (m/z): 520 (M^+^, 24.7%). Anal. Calcd. for C_32_H_24_N_8_: C, 73.83; H, 4.65; N, 21.52; Found: C, 73.77; H, 4.72; N, 21.51%.

### Comparison between conventional, grinding and microwave methods

The conventional, grinding and microwave reaction times were showed in Table 1. The yield economy (YE) was used as a term to determine the conventional, grinding, and microwave synthetic different efficiencies of the same reaction. Calculation of YE was occurred through: $E= \frac{yield\mathrm{\%}}{Reaction\,time "min"}$. YE was used to provide the yields obtained conclusively enhanced under the conventional, grinding and microwave conditions.$\mathrm{RME }=\frac{Wt\,of\,isolated\,product }{Wt\,of\,reactants}$. OE was used for the direct comparisons between the two reaction types and can be calculated through$OE= \frac{RME}{AE} x 100$. So, the yield economy (YE) can be considered as a metric to enhancing the conversion efficiencies of these two different synthetic methods of the same reaction. The reaction theoretical maximum efficiency was represented by using AE, while, RME gives the observed mass efficiency. The grinding and microwave reactions atom economy (AE) have the same values due to using two different reaction conditions to obtain the same desired compounds, as shown in Table 1.

**Table 1:** Show the comparison in terms of physical data between the synthesized compounds under grinding and microwave techniques

		2	3	4	5	6	7	8	9
Melting point (^o^C)	Found	100–102	122–124	172–174	140–142	104–106	222–224	138–140	160–162
	Lit*	100–102	120–122	170–172	140–142	106–108	220–222	138–140	160–162
Time “min”	Con	240	240	180	180	180	180	240	420
	G	10	12	14	17	16	13	11	14
	M.W	2	2.5	2	4	3	2.5	3.5	3
Yield %	Con	80	75	78	76	76	75	81	85
	G	89.4	89.1	88.9	87.3	88.7	86.0	89.5	90.8
	M.W	96.7	94.5	95.5	94.1	93.3	96.6	95.2	97.4
YE	Con	0.3333	0.3125	0.4333	0.4222	0.4222	0.4167	0.3375	0.2024
	G	8.94	7.43	6.35	5.14	5.54	6.62	7.50	6.49
	M.W	48.35	37.80	47.75	23.53	31.10	38.64	27.20	32.47
RME	Con	66.19	62.62	58.78	67.66	60.60	58.93	73.16	66.27
	G	73.97	74.39	66.99	77.73	70.72	67.57	80.83	76.03
	M.W	80.01	78.90	71.96	83.78	74.39	75.90	85.99	81.56
OE	Con	0.6619	0.6262	0.6562	0.7627	0.6400	0.6207	0.7634	0.6884
	G	0.7400	0.7439	0.7478	0.8762	0.7469	0.7117	0.8434	0.7898
	M.W	0.8001	0.7890	0.8033	0.9444	0.7856	0.7995	0.8972	0.8473
AE		100	100	89.58	88.71	94.69	94.94	95.84	96.26

*Lit. literature value, YE yield economy, RME reaction mass efficiency, OE optimum efficiency, AE atom economy, *Con*. conventional, *G*. grinding, *M.W*. microwave

### In vitro* anticancer evaluation*

#### Cell line

Hepatocellular carcinoma (HepG2), and colorectal carcinoma (HCT-116). The cell lines were obtained from ATCC via Holding company for biological products and vaccines (VACSERA), Cairo, Egypt.

#### Chemical reagents

The reagents RPMI-1640 medium, MTT and DMSO (sigma co., St. Louis, USA), Fetal Bovine serum (GIBCO, UK). 5-Fluorouracil was used as a standard anticancer drug for comparison.

#### MTT assay (1)

HepG2 and (HCT-116 were used to determine the inhibitory effects of compounds on cell growth using the MTT assay. The relative cell viability in percentage was calculated as (A570 of treated samples/A570 of untreated sample) X 100.

### ***Preparation of [***^***131***^***I]iodo-pyrazole derivative***

Radioiodination of compound **2** was performed using different values of the synthesized pyrazole derivative (25–400 µg) solutions, then 200 µL of chloramine-T (CAT) solution -freshly prepared- containing (100–600 µg). Then, 10 µL of (4 MBq) [^131^I]NaI was dropped to the reaction mixture, after that pH values were studied in a range from 4 to 9. The mixture was kept at ambient temperature for 24 h.

### Assessment of radiochemical purity

Formation of [^131^I]iodo-pyrazole derivative was assessed using TLC in a mobile phase chloroform: methanol (3: 1). 5 µL from reaction was added at the spotted point at the lower edge of the paper strip, then in ascending manner TLC was developed prepared using chloroform: methanol (3: 1 v/v). [^131^I] iodo-pyrazole derivative was migrate to R_f_ = 0.9; while free iodide remained at the spotted area. Then RCP was calculated.

### In-vivo evaluation of [^131^I]iodo-pyrazole derivative

Biodistribution studies was performed under the Animal Ethics Committee criteria, EAEA [45–47]. Swiss Albino mice aged three months weight 20–25 g were taken as a gift from the animal house of Egyptian Atomic Energy Authority. Ehrlich-Ascites-Carcinoma cell line (EAC) was taken from a female mouse mammary carcinoma, then used for induction of the tumour [34, 48]. 50 µL from diluted EAC cell line was injected intramuscularly in the right Albino swiss mouse thigh 5 days. Six groups of three mice each were injected intravenously at the mice tail. The substrate concentration (compound 2) in the intravenous injection was 6.14 × 10^–4^ mM, while the injected activity in each mice equal 0.4 MBq ([^131^I]NaI). Then the mice were anaesthetized with isoflurane, weighted and sacrificed using cervical dislocation method at different time intervals (0.25, 0.5, 1, 2, 3, and 24 h) and the organs were dissected, weighed and counted. Then results were calculated as % ID/g.

## Results and discussion

### Synthesis

**Scheme 2:**
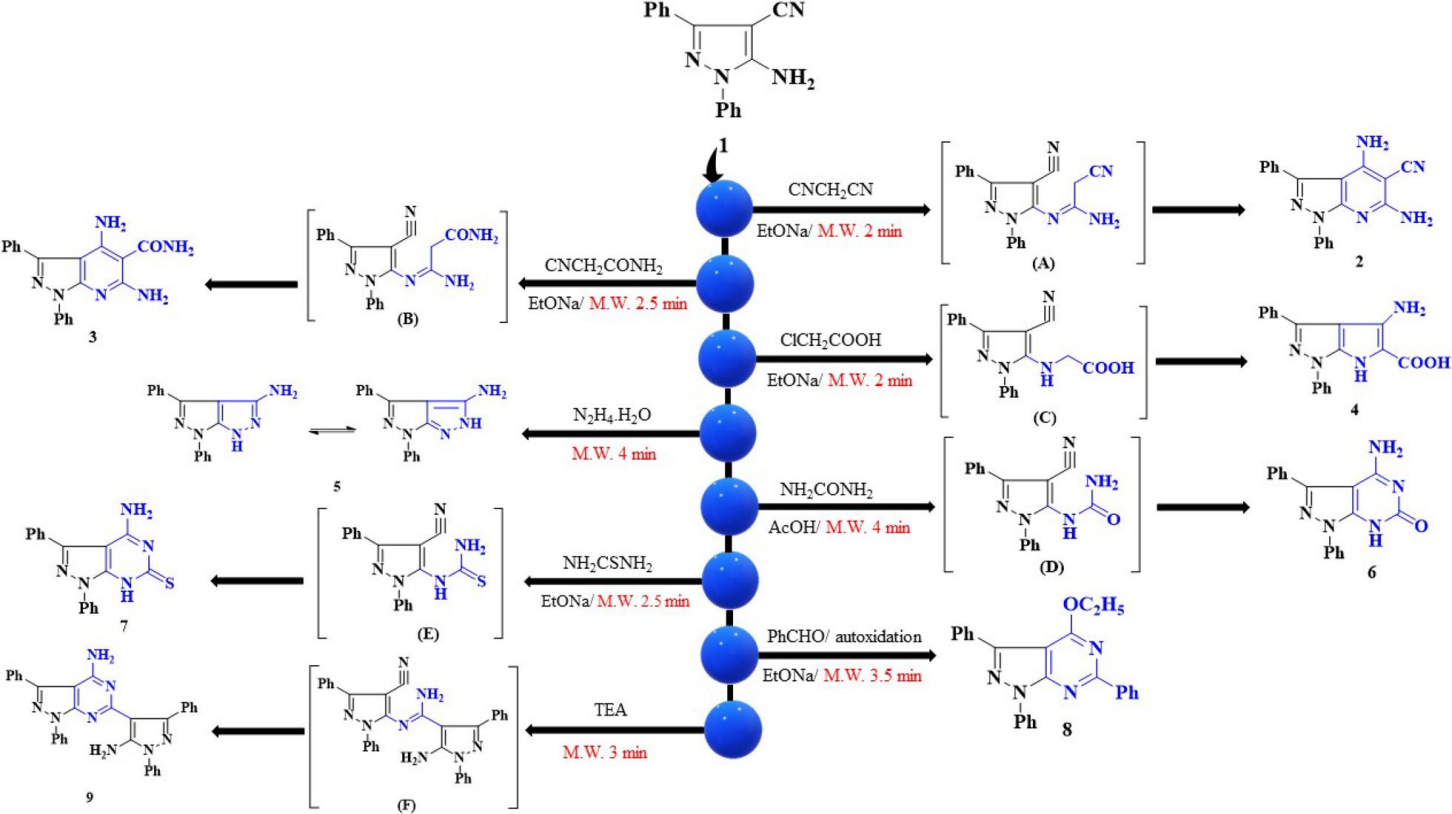
Synthesis of pyrazolopyridine (2, 3), pyrazolopyrrole (4), pyrazolopyrazole (5), and pyrazolopyrimidine (6–9) derivatives

Microwave and grinding techniques were used for heterocyclic synthesis to obtain an environmentally friendly methods are in accordance with the green chemistry principles [49–51]. Our objective was to develop a synthetic method for the reaction of 5-amino-1,3-diphenyl-1H-pyrazole-4-carbonitrile (1) with different nucleophilic and electrophilic reagents using microwave and grinding techniques. The reactions were repeated using conventional technique with the similar outcome. The bifunctional starting material 1 can be used as a key intermediate in interesting bicyclic compounds synthesis, via its irradiation with different nucleophilic reagents namely malononitrile, cyanoacetamide and/or chloroacetic acid in presence of sodium ethoxide producing pyrazolopyridine and pyrazolopyrrole derivatives 2–4, respectively. Formation of di-enaminonitrile derivative 2 occurred through the condensation reaction between the amino group and malononitrile to form the intermediate (A), which undergo ring closure formed the expected compound. The structure of compound 2 was confirmed from its IR spectrum, which displayed bands at 3442, 3315, 3290 cm^−1^ characteristics for the NH_2_ group. Its ^1^H-NMR spectrum showed a peak corresponding to 2NH_2_ at 10.32 ppm. Its mass spectrum revealed a molecular ion peak at 326 (M^+^) corresponding to C_19_H_14_N_6_. However, formation of compound 3 may takes place through the nucleophilic attack of the amino group on the cyano group of cyanoacetamide to form the intermediate (B), which underwent ring closure to produce the expected derivative. While the structure of compound 3 was supported from its IR spectrum, which was devoid of CN band and displayed band at 1658 cm^−1^ characteristics for the C = O group. Its ^1^H-NMR spectrum display peaks corresponding to 2NH_2_ and CONH_2_ at 8.89 and 11.24 ppm, respectively. Its ^13^C-NMR spectrum showed a peak corresponding to C = O at 162.3 ppm. Its mass spectrum revealed a molecular ion peak at 344 (M^+^) corresponding to C_19_H_16_N_6_O. Also, in case of compound 4 formation, chloroacetic acid reacted with the amino group forming the intermediate (C) through the elimination of one HCl molecule and then ring closure takes place. Also, IR spectrum of compound 4 showed disappearance of CN band in its IR spectrum and displayed bands at 3223 and 1709 cm^−1^ characteristics for the NH and C = O groups, respectively. Its ^1^H-NMR spectrum display peaks at 5.08, 10.00 and 10.40 ppm corresponding to NH_2_, NH and OH, respectively. Its mass spectrum revealed a molecular ion peak at 318 (M^+^) corresponding to C_18_H_14_N_4_O_2_. Furthermore, compound 1 was subjected to the reaction with hydrazine hydrate in microwave to produce pyrazolopyrazole derivative 5 (Scheme 2). Furthermore, the structure of compound 5 was illustrated from its IR spectrum, which was devoid of CN band and revealed bands at 3385, 3313 and 3201 specifics for NH_2_ and NH groups, respectively. Its ^1^H-NMR spectrum display peaks at 8.68 and 10.31 ppm corresponding to NH and NH_2_, respectively. Its mass spectrum revealed a molecular ion peak at 275 (M^+^) corresponding to C_16_H_13_N_5_.

While, pyrazolopyrimidin-6-one 6 was obtained through the condensation reaction between the amino group and urea to form the intermediate (D) through the elimination of one molecule of ammonia which undergo ring closure formed the expected compound. However, the structure of compound 6 was illustrated from its IR spectrum, which was devoid of CN band and revealed band at 1688 specifics for C = O group. Its ^1^H-NMR spectrum display peaks at 9.55 and 10.28 ppm corresponding to NH_2_ and NH, respectively. Its ^13^C-NMR spectrum showed a peak corresponding to C = O at 159.8 ppm. Its mass spectrum revealed a molecular ion peak at 303 (M^+^) corresponding to C_17_H_13_N_5_O. Pyrazolopyrimidin-6-thione 7 was furnished in the same manner through the intermediate (E). While the structure of compound **7** was supported from its IR spectrum, which was devoid of CN band and displayed band at 3200 and 1287 corresponding to NH and C = S groups, respectively. Its ^1^H-NMR spectrum display peaks at 10.33 and 13.14 ppm corresponding to NH_2_ and NH, respectively. Its ^13^C-NMR spectrum showed a peak corresponding to C = S at 189.1 ppm. Its mass spectrum revealed a molecular ion peak at 319 (M^+^) corresponding to C_17_H_13_N_5_S. Noteworthy, pyrazolopyrimidine derivative 8 was formed through the compound 1 irradiation with benzaldehyde in the presence of sodium ethoxide. Furthermore, the structure of compound 8 was illustrated from its ^1^H-NMR spectrum display peaks at 1.31 and 4.20 corresponding to CH_3_ and CH_2_ groups, respectively. Its ^13^C-NMR spectrum showed a peak corresponding to CH_3_ and CH_2_ at 15.9 and 56.8 ppm, respectively. Its mass spectrum revealed a molecular ion peak at 392 (M^+^) corresponding to C_25_H_20_N_4_O. However, compound 9 formation takes place through the two molecules of compound 1 condensation, where the first molecule amino group attacks the second molecule cyano group, producing the intermediate (F) followed by ring closure (Scheme 2). However, the structure of compound 9 was illustrated from its ^1^H-NMR spectrum display peaks at 9.41 and 10.57 ppm corresponding to 2NH_2_ groups. Its mass spectrum revealed a molecular ion peak at 520 (M^+^) corresponding to C_32_H_24_N_8_.

### In-vitro anticancer evaluation

The synthesized pyrazole derivatives were *in-vitro* examined for its cytotoxicity against two types of human cancer cells (HepG2 and HCT-116) as shown in Table 2. their cytotoxic ability was compared with 5-Fu as a reference. Results showed that the tested compound 2 has the highest cytotoxic effect against the two assessed cell lines showing an obvious cytotoxic activity. The obtained IC_50_ values of compound 2 were 9.2 ± 2.8 and 7.7 ± 1.8 µM against HepG2 and HCT-116, respectively.

**Table 2:** Anticancer activity of the synthesized pyrazole derivatives against HepG2 and HCT-116 cell lines

Comp	*In-vitro* Cytotoxicity IC_50_ (µM)^a^	
	HepG2	HCT-116
5-Fu^**b**^	7.86 ± 0.5	5.35 ± 0.3
2	9.2 ± 2.8	7.7 ± 1.8
3	22.8 ± 2.1	29.4 ± 2.3
4	14.5 ± 2.8	15.2 ± 2.4
5	8.8 ± 5.1	13.8 ± 3.9
6	91.4 ± 4.2	51.0 ± 2.7
7	10.2 ± 2.1	13.1 ± 2.3
8	12.1 ± 5.1	17.4 ± 3.9
9	55.7 ± 2.7	33.6 ± 2.1

^**a**^IC_50_ (µM): 1–10 (very strong). 11–20 (strong). 21–50 (moderate). 51–100 (weak) and above 100 (non-cytotoxic)

^**b**^5-Fu: 5-Fluorouracil

### Structure–Activity Relationship (SAR)

In this study, a series of heterocyclic bearing Pyrazole moiety derivatives were synthesized and their cytotoxicity were evaluated against two cancer cell lines (HepG2 and HCT-116). Various derivatives were synthesized from 5-amino-1,3-diphenyl-1H-pyrazole-4-carbonitrile (compound 1). The tested compounds results showed that exhibited very strong, strong, moderate, or weak anticancer activities against the tested cell lines. First, malononitrile was added forming pyridine moiety having diamino and cyano group (compound 2) appeared to have the highest biological activity against both cell lines. Further modifications were implemented which resulted in drastic decrease in activity. The activity of compounds 2 may be related to the core of compound which is pyrazolopyridine ring in addition to the cyano group presence at position 5 of the pyridine ring which is an electron-withdrawing group, in addition to the two amino groups presence at positions 4 and 6 of compound 2. However, the addition of hydrazine hydrate having another pyrazole moiety (compound 5) caused slight decrease in the cytotoxic activity against HCT-116 and slight increase against HepG. While the activity of compounds 5 may be related to the pyrazolopyrazole ring in addition to the two amino groups presence at positions 3 and the NH of the ring of compound 5. However, the activity of compounds 7 may be related to pyrazolopyrimidine ring in addition to the C = S group presence at position 7 of the pyrimidine ring, which is a rich by electron centres, in addition to the two amino groups presence at position 4 of compound 7. Further modifications using cyanoacetamide, chloroacetic acid, thiourea, benzaldehyde, and TEA caused some decrease in the cytotoxic activity. (Additional file 1).

### Radioiodination of pyrazole derivative

**Fig. 1:**
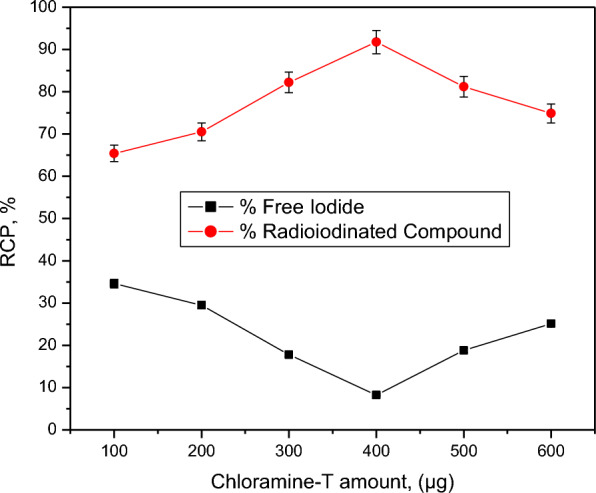
Effect of CAT amount on RCP of [^131^I]I-compound 2. Reaction conditions: 100 µg compound 2, pH 6, 10 µL (3.7 MBq) [^131^I] NaI solution for 30 min at ambient temperature, *n* = 3 independent experiments

**Fig. 2:**
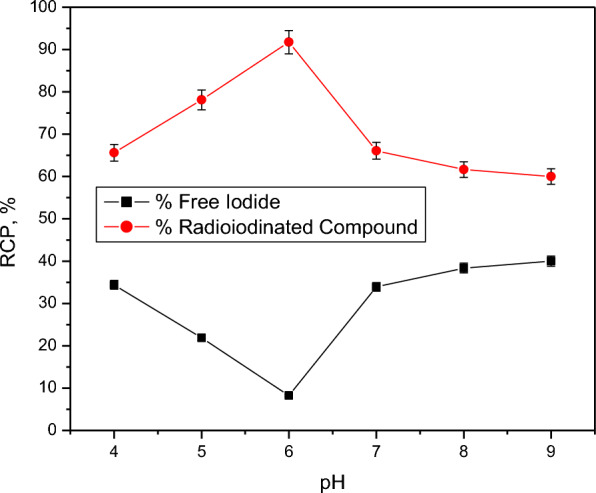
Effect of pH amount on RCP of [^131^I]I-compound 2. Reaction conditions: 400 µg CAT, 100 µg compound 2, 10 µL (3.7 MBq) [^131^I] NaI solution for 30 min at ambient temperature, *n* = 3 independent experiments

**Fig. 3:**
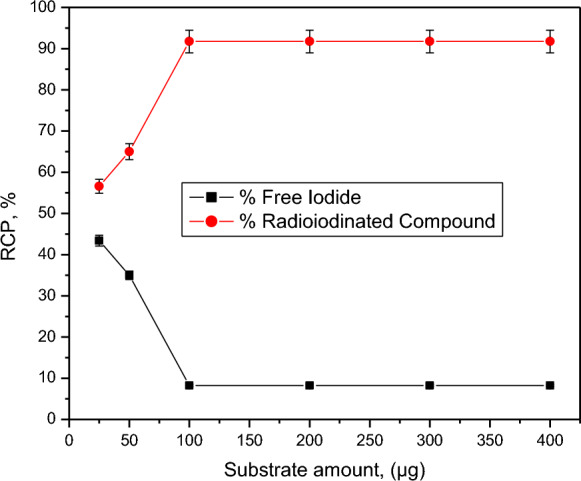
Effect of compound 2 amount on RCP of [^131^I]I-compound 2. Reaction conditions: 400 µg CAT, pH 6, 10 µL (3.7 MBq) [^131^I]NaI solution for 30 min at ambient temperature, *n* = 3 independent experiments

**Fig. 4:**
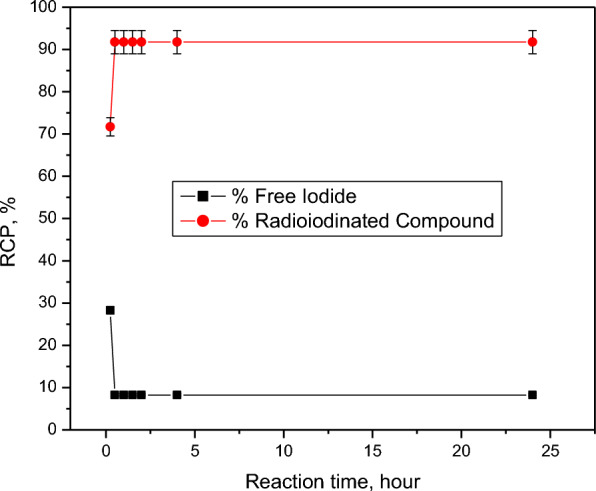
Effect of reaction time on RCP of [^131^I] I-compound 2. Reaction conditions: 400 µg CAT, 100 µg compound 2, pH 6, 10 µL (3.7 MBq) [^131^I] NaI solution at ambient temperature, *n* = 3 independent experiments

Compound 2 can be used as a chemotherapeutic agent because it is the highest cytotoxic synthesized compound against both HepG2 and HCT-116 cell lines. Compound 2 was radiolabeled with iodine-131 to evaluate its biodistribution in animal models and also to be studied to carry the therapeutic radioisotope (iodine-131) to the target site (cancer tissues) for targeted radio therapy (TRT). The highest radiochemical purity of radioiodinated compound 2 was obtained by optimization of all parameters affecting the radioiodination process. The electrophilic substitution of iodine-131 was carried out in the presence of an oxidizing agent (chloramine-T). Chloramine-T, which converts iodide ion to iodonium ion [52], has a clear impact on the process of radioiodination, also an electrophilic substitution was happen by the permission from the oxidizing agent. 400 µg from CAT was the optimum amount required to create the highest RCP of 91.74% (Fig. 1). Different CAT amounts may cause undesirable oxidative byproduct and Insufficient oxidation of I-131 by increasing or decreasing of its value, respectively [53]. pH value is a main factor which affect the RCP. The RCP was decreased in acidic media and increased around neutral values then decreases again towards basic media (Fig. 2). The formation of hypoiodite (IO^−^) ion and iodate (IO_3_^−^) ions may be the cause of this decrease in RCP value [54]. Figure 3 illustrate the effect of compound 2 amount on the RCP. The maximum RCP was obtained at 100 µg from compound 2, this amount can react with all ions of iodonium in the reaction. By increasing the amount of substrate, the RCP still stable around the highest yield. Figure 4 showed the time effect on the radioiodination process which indicates its fastness. The reaction stability study was done for 24 h.

### Biodistribution study of [^131^I]iodo-pyrazole derivative

**Fig. 5:**
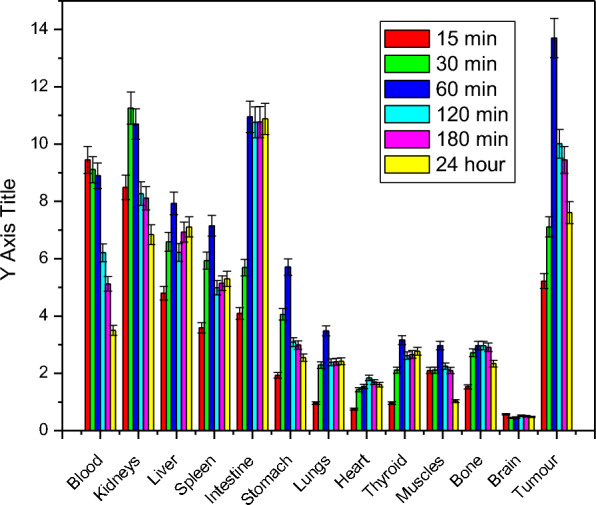
Biological distribution of [^131^I] I- compound 2 in solid tumor-bearing mice. *n* = 3 independent experiments

**Fig. 6:**
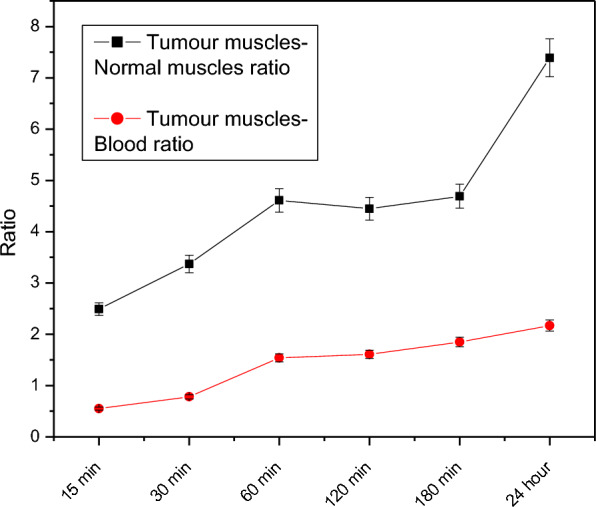
T/NT of [^131^I] I- compound 2 in solid tumour-bearing mice (Tumour muscles/Normal muscles, and Tumour muscles/Blood). *n* = 3 independent experiments

The biodistribution data of [^131^I]I- compound **2** (Fig. 5) in tumour-bearing mice at different times (0.25, 0.5, 1, 2, 3, and 24 h) post-injection reveals that excretion of [^131^I]I-compound 2 occurs via both urinary and hepatobiliary pathways due to relatively high uptake for kidney and liver over the time. As shown in Fig. 5 [^131^I]I-compound 2 was rapidly distributed for different body organs as a result of rapid clearance from the blood. There is no specific accumulation of radioactivity in the different organs over the time except tumour site. Regarding tumour uptake; [^131^I]I-compound 2 was gathered in a high ratio reached 13.7 after one hour in comparison with 2.97 at normal muscle at the same time point. Figure 6 shows the target non-target ratios between tumour muscles and normal muscles, and between tumour muscles and blood. This figure declares that one hour time post-injection may be the best time point to use [^131^I]I- compound 2 as a theranostic agent.

## Conclusion

Pyrazole-based analogs play a significant role in the creation of numerous medications and have a wide spectrum of pharmacological actions. Pyrazole derivatives were synthesized via green methods to be tested for *in-vitro* chemo- and TRT. These substances were synthesized as a result of their noteworthy biological activities, which have made them prospective candidates in the discovery and development of new drugs. The most effective synthesized pyrazole derivative against the two tested cancer cell lines is 4,6-Diamino-1,3-diphenyl-1H-pyrazolo[3,4-b]pyridine-5-carbonitrile which shows high effective action against both HepG2 and HCT-116 cell lines and also it has high accumulation at cancer site which guide us to conclude that this derivative (compound 2) could be used as cancer chemotherapeutic agent. Also, radioiodination of this compound using I-131 -which is used as therapeutic radioisotope- making this compound to be used for TRT. In conclusion the synthesized new pyrazole derivative can be used for chemo/radioisotope therapy after further preclinical studies.

### Supplementary Information

**Additional file 1. Figure S1. **IR spectrum of compound 2. **Figure S2.** ^1^H-NMR of compound 2. **Figure S3.** MS of compound 2. **Figure S4.** IR spectrum of compound 3. **Figure S5.** ^1^H-NMR of compound 3. **Figure S6.** ^13^C-NMR of compound 3. **Figure S7.** MS of compound 3. **Figure S8.** IR spectrum of compound 4. **Figure S9.** ^1^H-NMR of compound 4. **Figure S10.** MS of compound 4. **Figure S11.** IR spectrum of compound 5. **Figure S12.** ^1^H-NMR of compound 5. **Figure S13.** MS of compound 5. **Figure S14.** IR spectrum of compound 6. **Figure S15.** ^1^H-NMR of compound 6. **Figure S16.** ^13^C-NMR of compound 6. **Figure S17.** MS of compound 6. **Figure S18.** IR spectrum of compound 7. **Figure S19.** ^1^H-NMR of compound 7. **Figure S20.** ^13^C-NMR of compound 7. **Figure S21.** MS of compound 7. **Figure S22.** IR spectrum of compound 8. **Figure S23.** ^1^H-NMR of compound 8. **Figure S24.** ^13^C-NMR of compound 8. **Figure S25.** MS of compound 8. **Figure S26.** IR spectrum of compound 9. **Figure S27.** ^1^H-NMR of compound 9. **Figure S28.** ^13^C-NMR of compound 9. **Figure S29.** MS of compound 9.

## Data Availability

All data generated or analyzed during this study are included in this published article and supplementary materials.
