# Supplementary material for: Green synthesis of highly functionalized heterocyclic bearing pyrazole moiety for cancer-targeted chemo/radioisotope therapy
Source: BMC Chem. 2023 Oct 18;17(1):139. doi: 10.1186/s13065-023-01053-7 (PMC10585773; doi:10.1186/s13065-023-01053-7)
Supplement: Supplementary file 1 — Additional file 1. Figure S1. IR spectrum of compound 2. Figure S2. 1H-NMR of compound 2. Figure S3. MS of compound 2. Figure S4. IR spectrum of compound 3. Figure S5. 1H-NMR of compound 3. Figure S6. 13C-NMR of compound 3. Figure S7. MS of compound 3. Figure S8. IR spectrum of compound 4. Figure S9. 1H-NMR of compound 4. Figure S10. MS of compound 4. Figure S11. IR spectrum of compound 5. Figure S12. 1H-NMR of compound 5. Figure S13. MS of compound 5. Figure S14. IR spectrum of compound 6. Figure S15. 1H-NMR of compound 6. Figure S16. 13C-NMR of compound 6. Figure S17. MS of compound 6. Figure S18. IR spectrum of compound 7. Figure S19. 1H-NMR of compound 7. Figure S20. 13C-NMR of compound 7. Figure S21. MS of compound 7. Figure S22. IR spectrum of compound 8. Figure S23. 1H-NMR of compound 8. Figure S24. 13C-NMR of compound 8. Figure S25. MS of compound 8. Figure S26. IR spectrum of compound 9. Figure S27. 1H-NMR of compound 9. Figure S28. 13C-NMR of compound 9. Figure S29. MS of compound 9. [file 13065_2023_1053_MOESM1_ESM.pdf]

Additional file 1

**Suppl. Fig. 1**	IR spectrum of compound 2
**Suppl. Fig. 2**	^{1}H-NMR of compound 2
**Suppl. Fig. 3**	MS of compound 2
**Suppl. Fig. 4**	IR spectrum of compound 3
**Suppl. Fig. 5**	^{1}H-NMR of compound 3
**Suppl. Fig. 6**	^{13}C-NMR of compound 3
**Suppl. Fig. 7**	MS of compound 3
**Suppl. Fig. 8**	IR spectrum of compound 4
**Suppl. Fig. 9**	^{1}H-NMR of compound 4
**Suppl. Fig. 10**	MS of compound 4
**Suppl. Fig. 11**	IR spectrum of compound 5
**Suppl. Fig. 12**	^{1}H-NMR of compound 5
**Suppl. Fig. 13**	MS of compound 5
**Suppl. Fig. 14**	IR spectrum of compound 6
**Suppl. Fig. 15**	^{1}H-NMR of compound 6
**Suppl. Fig. 16**	^{13}C-NMR of compound 6
**Suppl. Fig. 17**	MS of compound 6
**Suppl. Fig. 18**	IR spectrum of compound 7
**Suppl. Fig. 19**	^{1}H-NMR of compound 7
**Suppl. Fig. 20**	^{13}C-NMR of compound 7
**Suppl. Fig. 21**	MS of compound 7
**Suppl. Fig. 22**	IR spectrum of compound 8
**Suppl. Fig. 23**	^{1}H-NMR of compound 8
**Suppl. Fig. 24**	^{13}C-NMR of compound 8
**Suppl. Fig. 25**	MS of compound 8
**Suppl. Fig. 26**	IR spectrum of compound 9
**Suppl. Fig. 27**	^{1}H-NMR of compound 9
**Suppl. Fig. 28**	^{13}C-NMR of compound 9
**Suppl. Fig. 29**	MS of compound 9

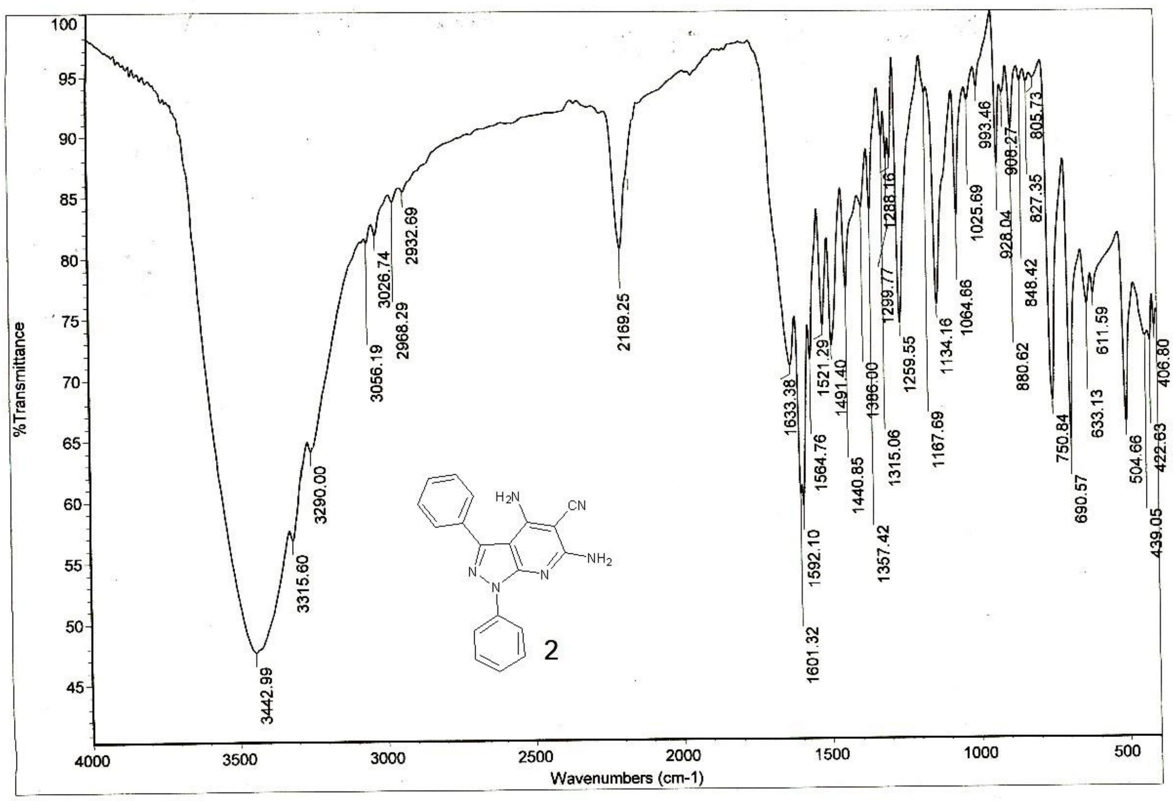

Suppl. Fig. 1 IR spectrum of compound 2

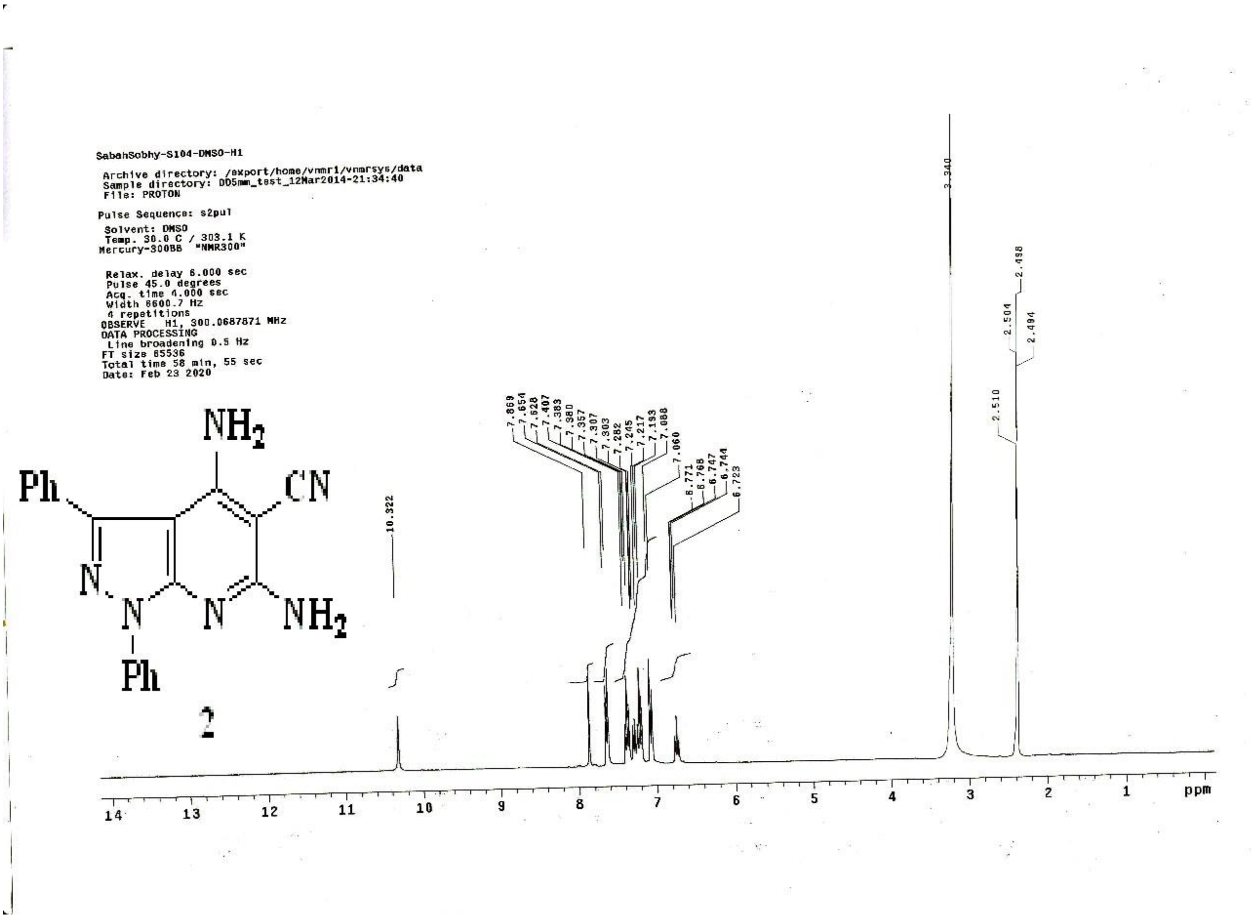

Suppl. Fig. 2 ^{1}H-NMR of compound 2

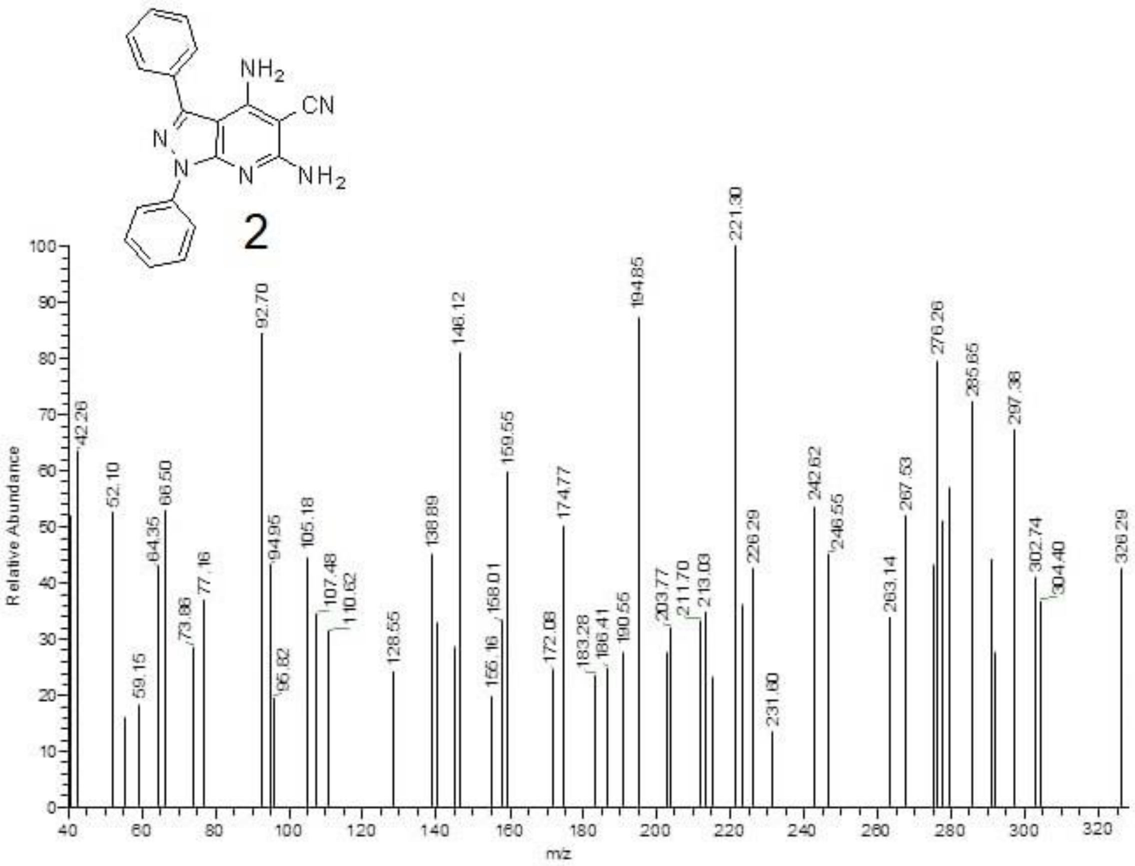

Suppl. Fig. 3 MS of compound 2

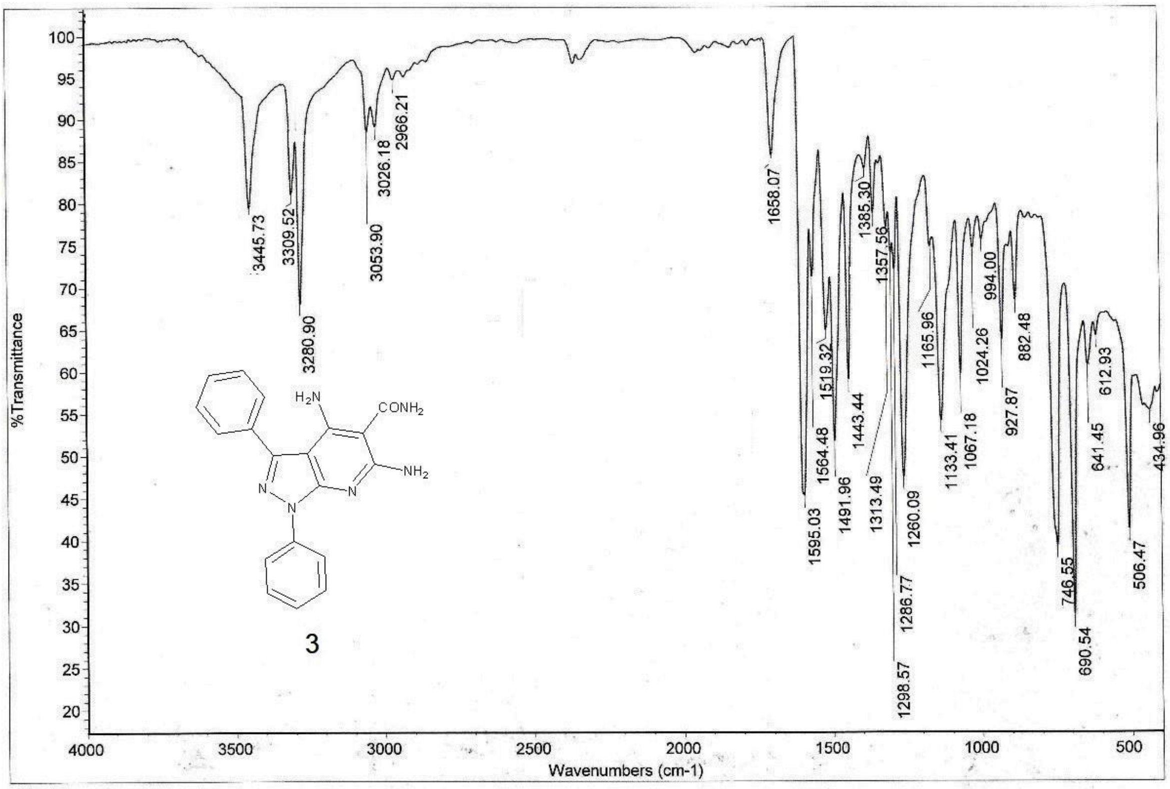

Suppl. Fig. 4 IR spectrum of compound 3

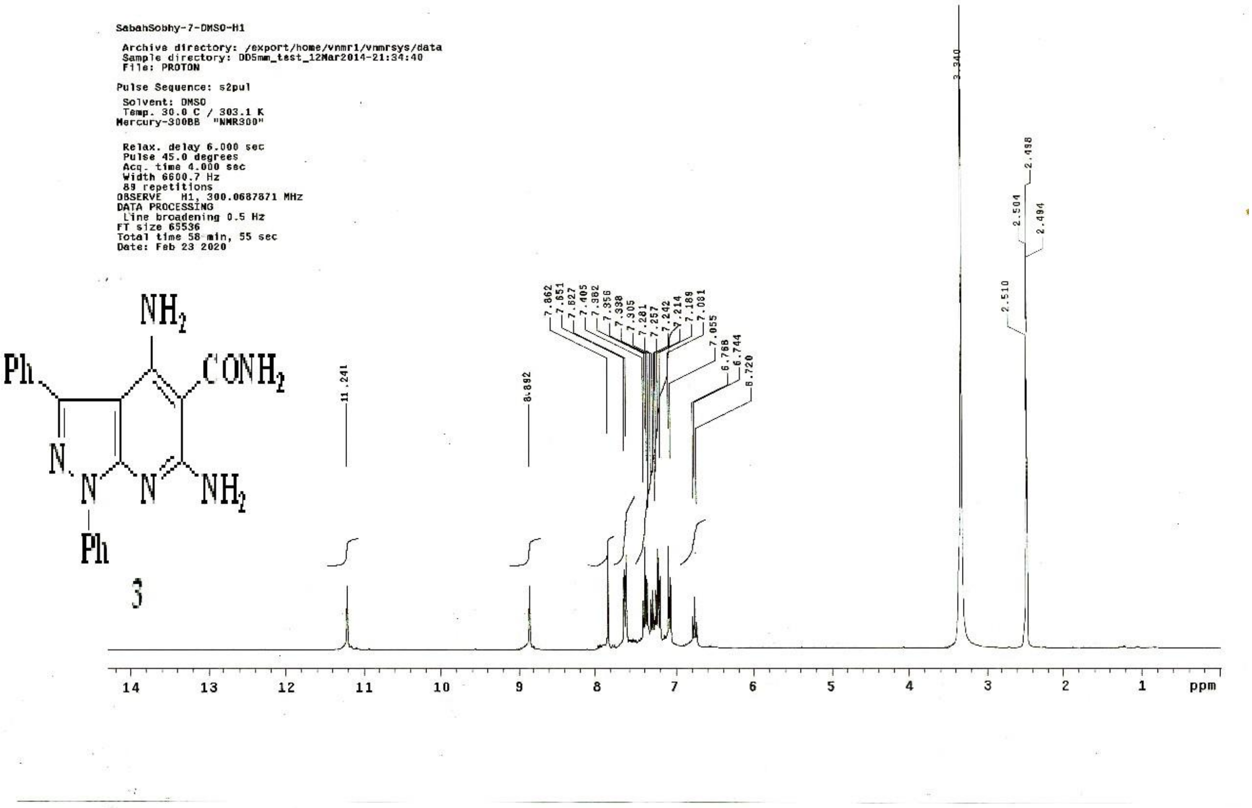

Suppl. Fig. 5 ^{1}H-NMR of compound 3

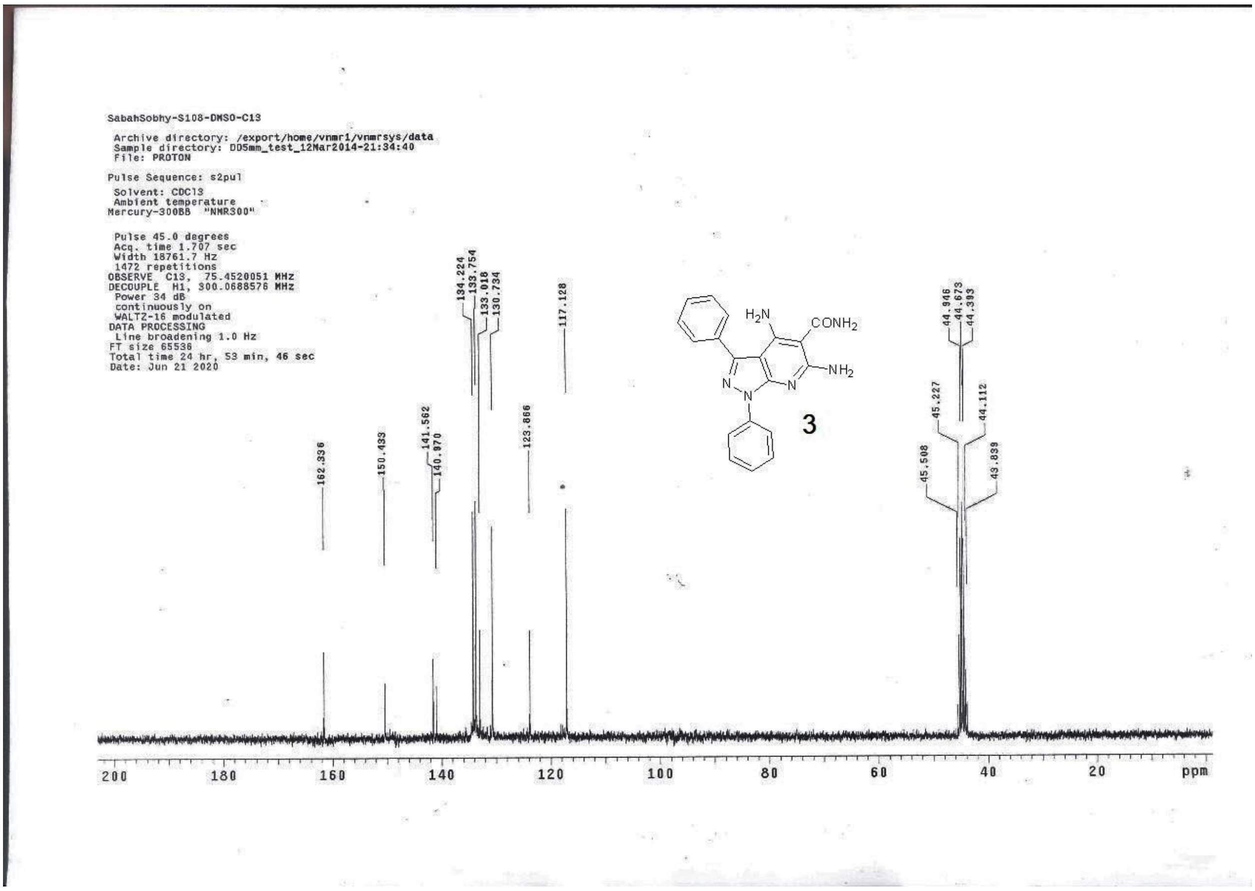

Suppl. Fig. 6 ^{13}C-NMR of compound 3

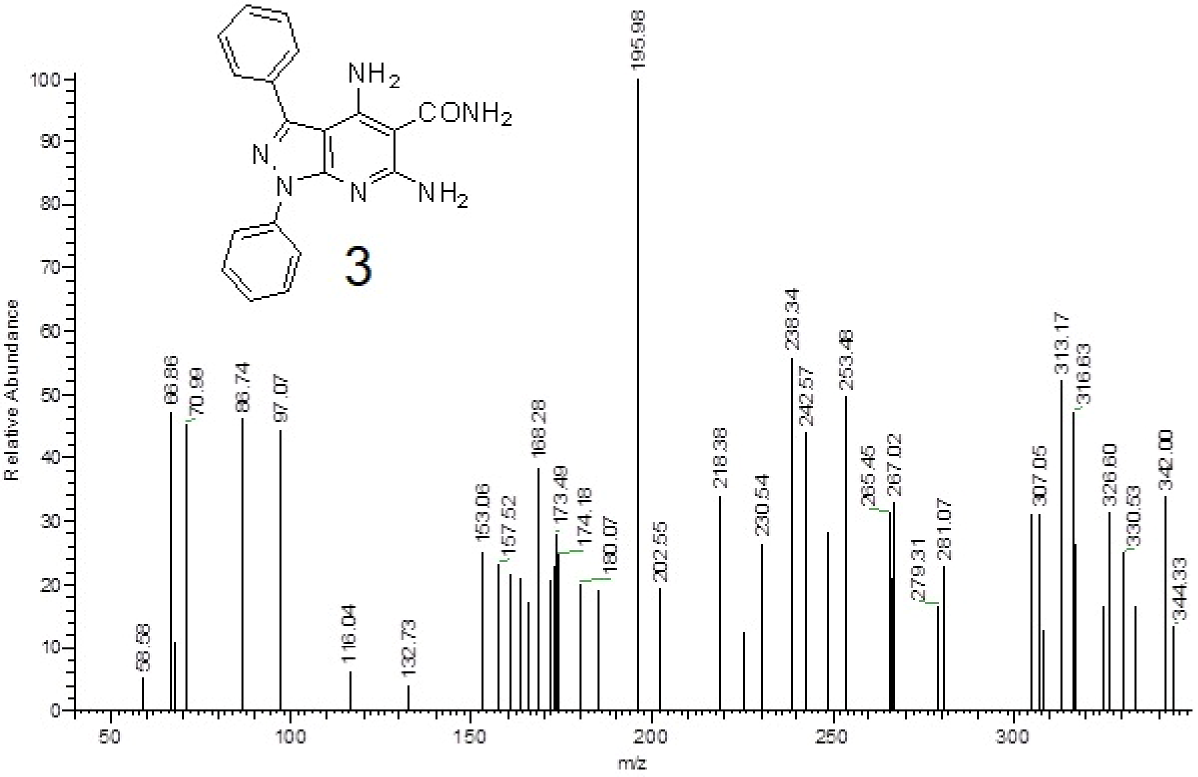

Suppl. Fig. 7 MS of compound 3

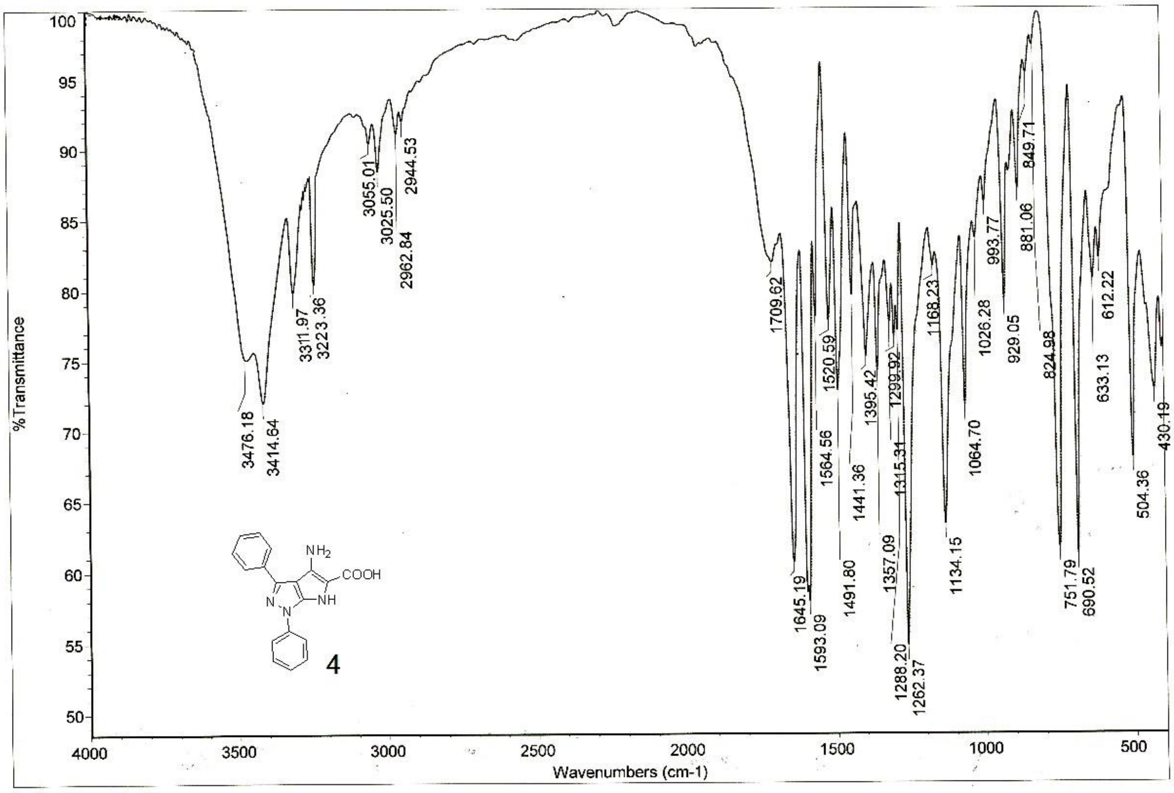

Suppl. Fig. 8 IR spectrum of compound 4

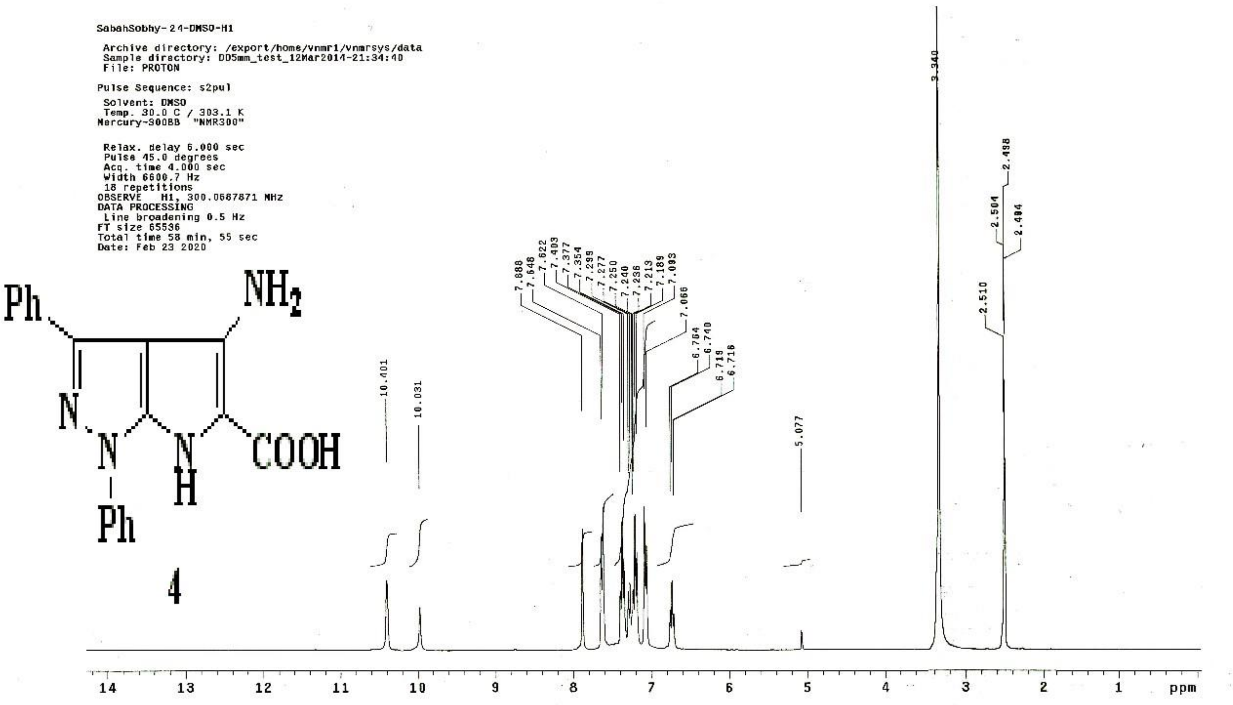

Suppl. Fig. 9 ^{1}H-NMR of compound 4

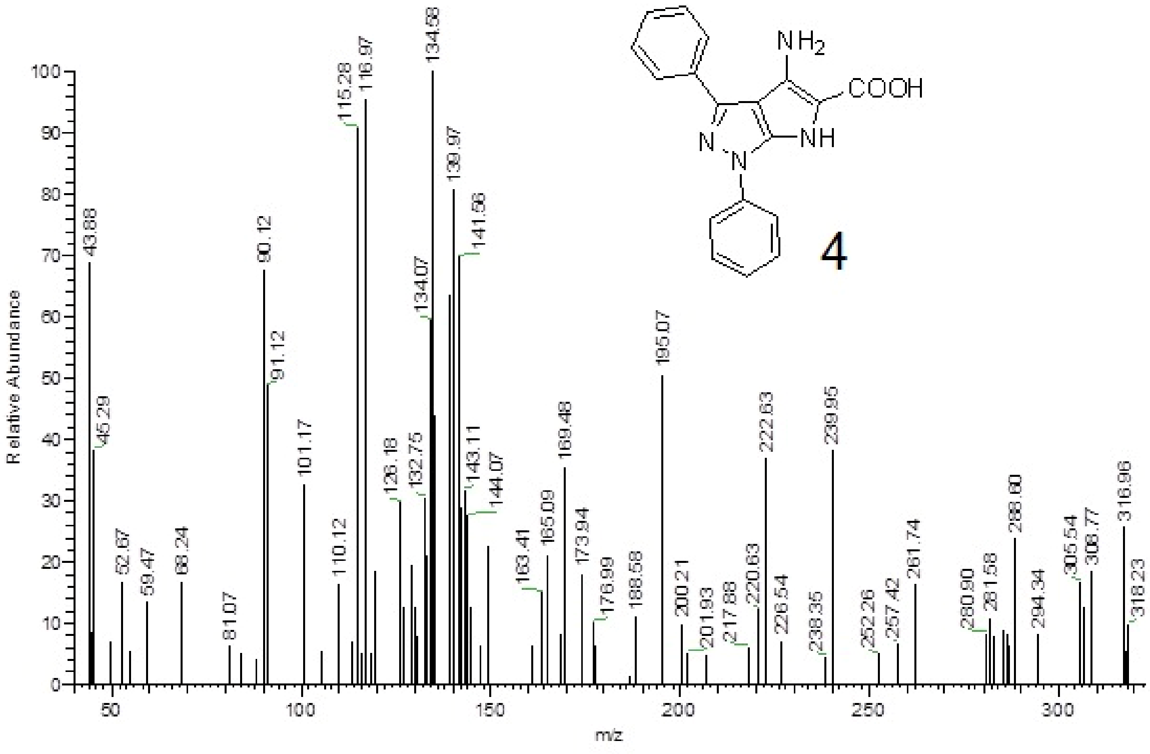

Suppl. Fig. 10 MS of compound 4

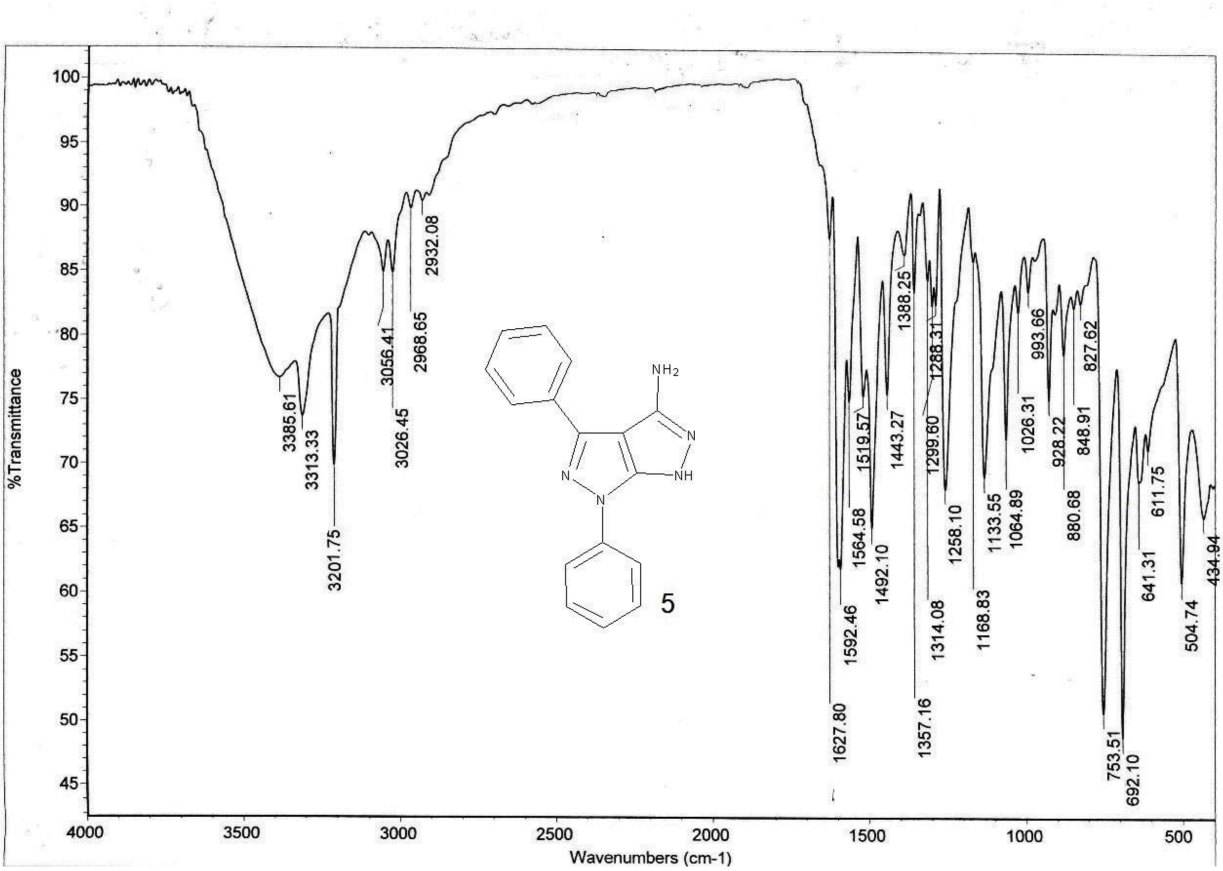

Suppl. Fig. 11 IR spectrum of compound 5

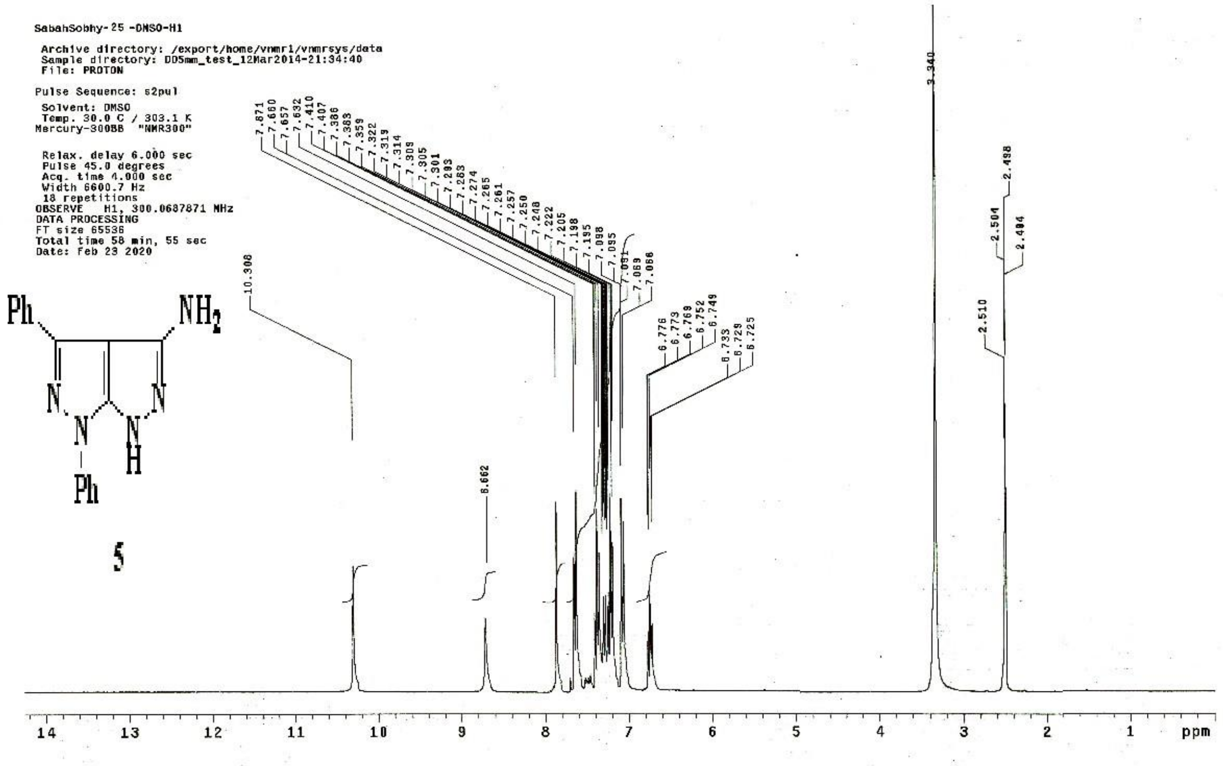

Suppl. Fig. 12 ^{1}H-NMR of compound 5

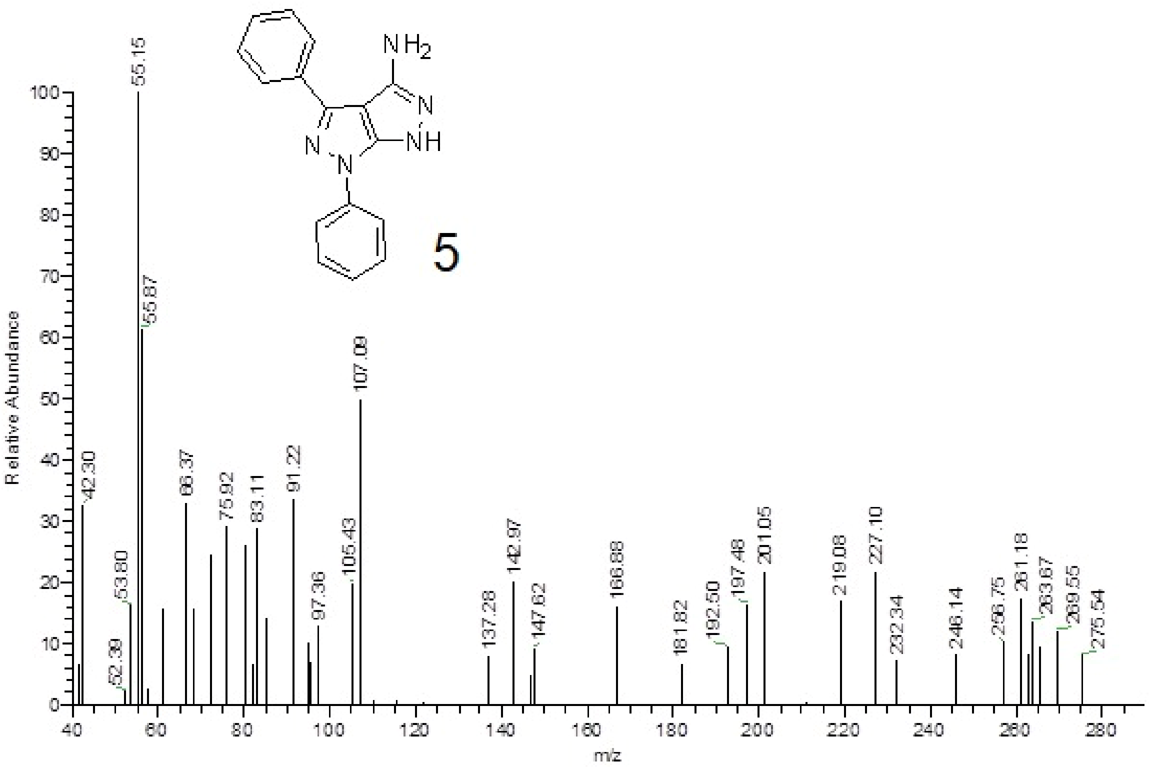

Suppl. Fig. 13 MS of compound 5

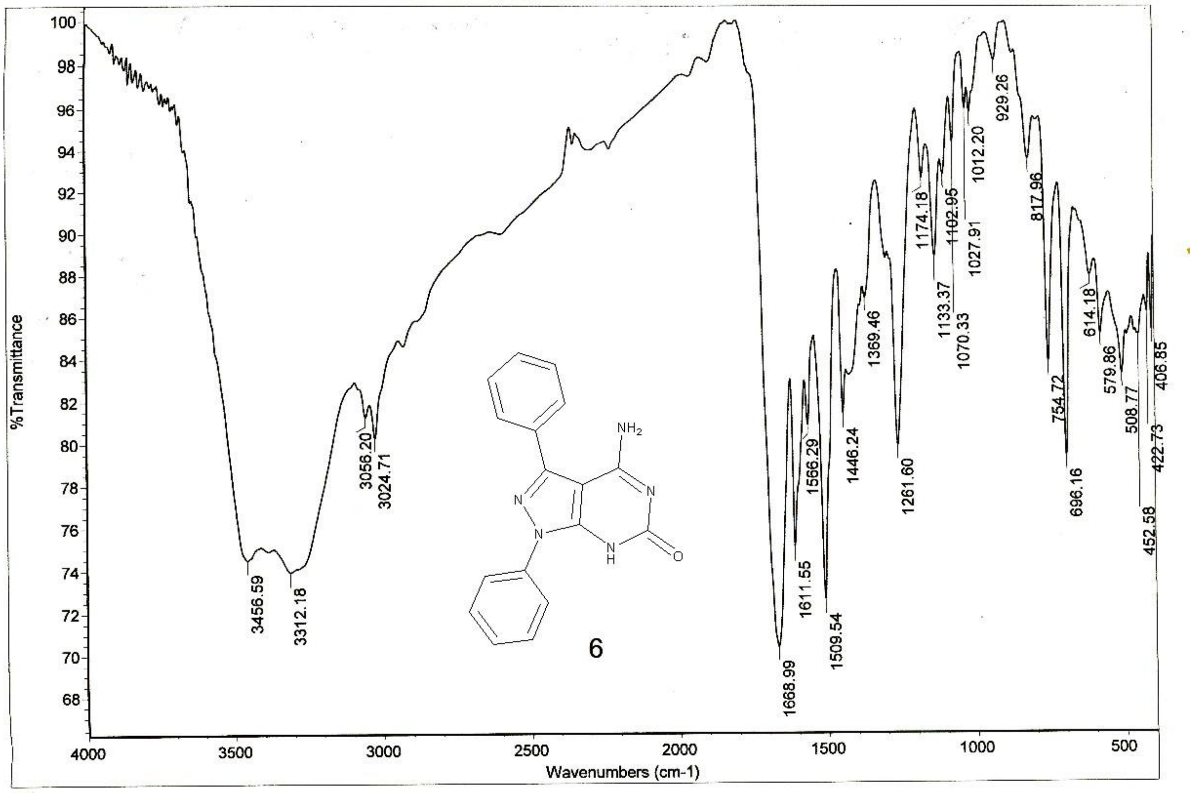

Suppl. Fig. 14 IR spectrum of compound 6

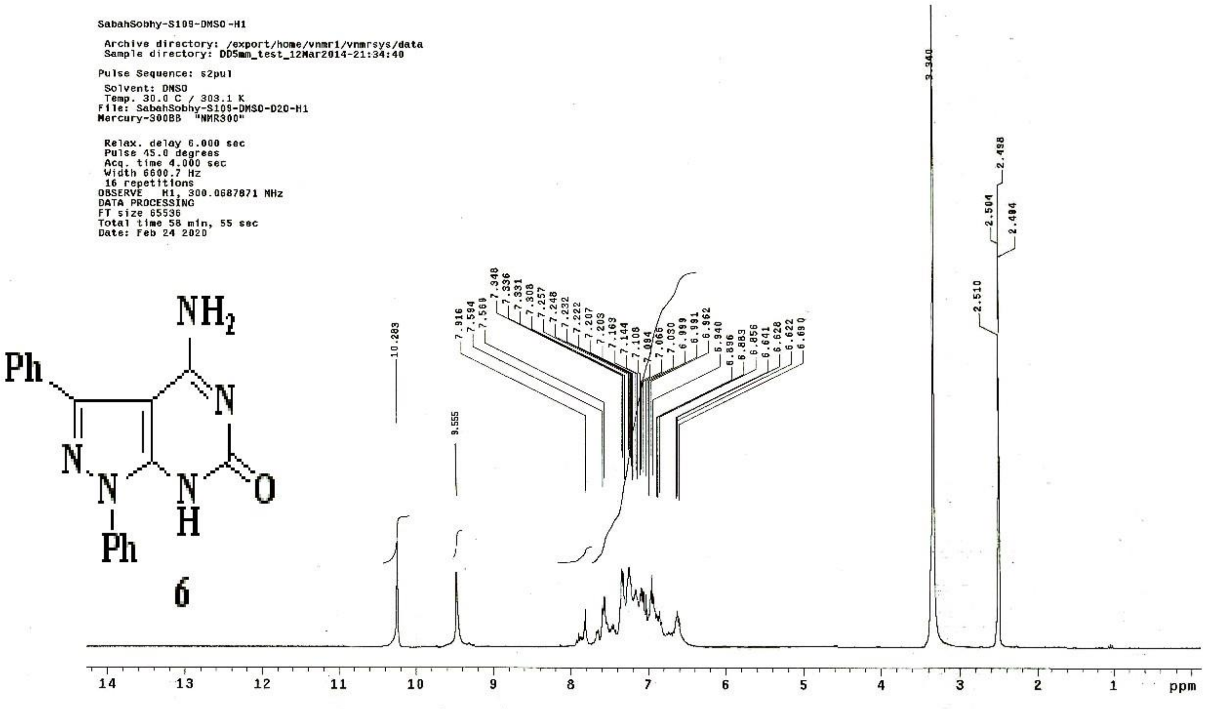

Suppl. Fig. 15 ^{1}H-NMR of compound 6

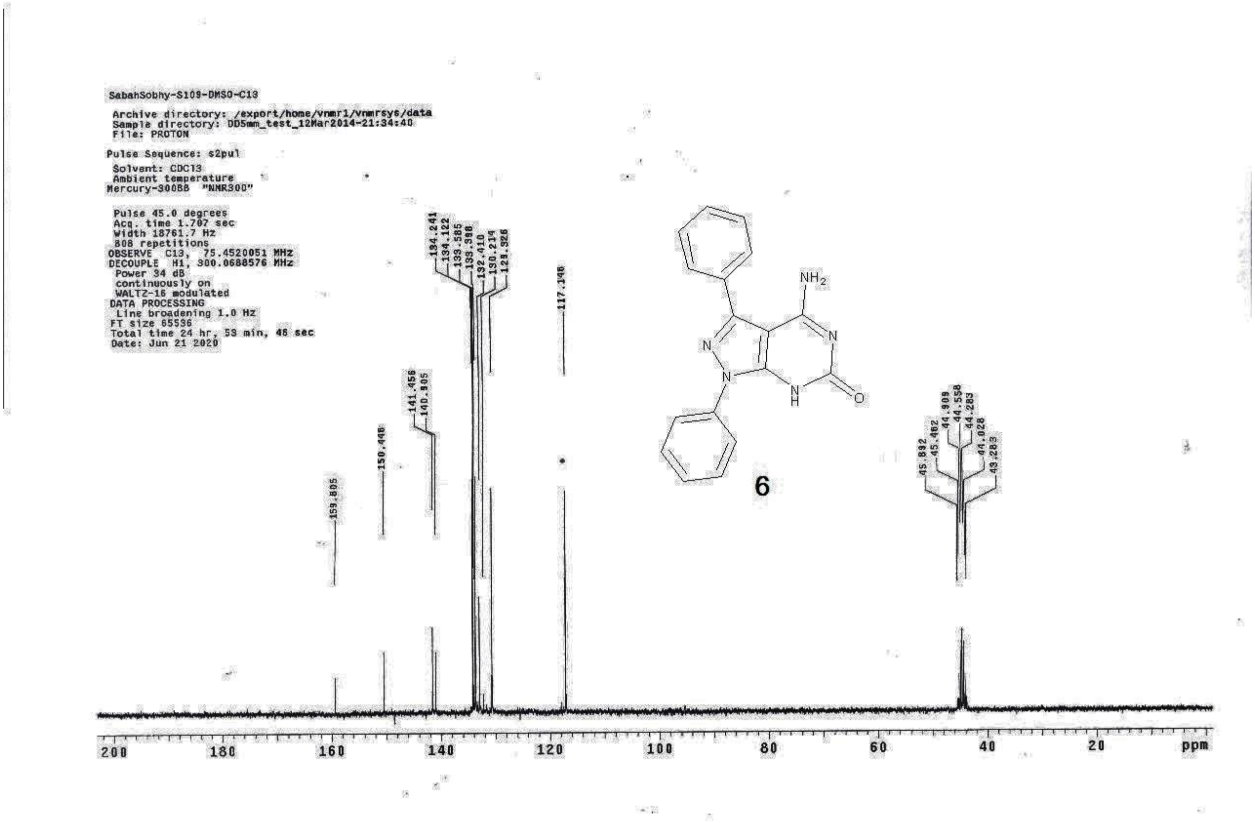

Suppl. Fig. 16 ^{13}C-NMR of compound 6

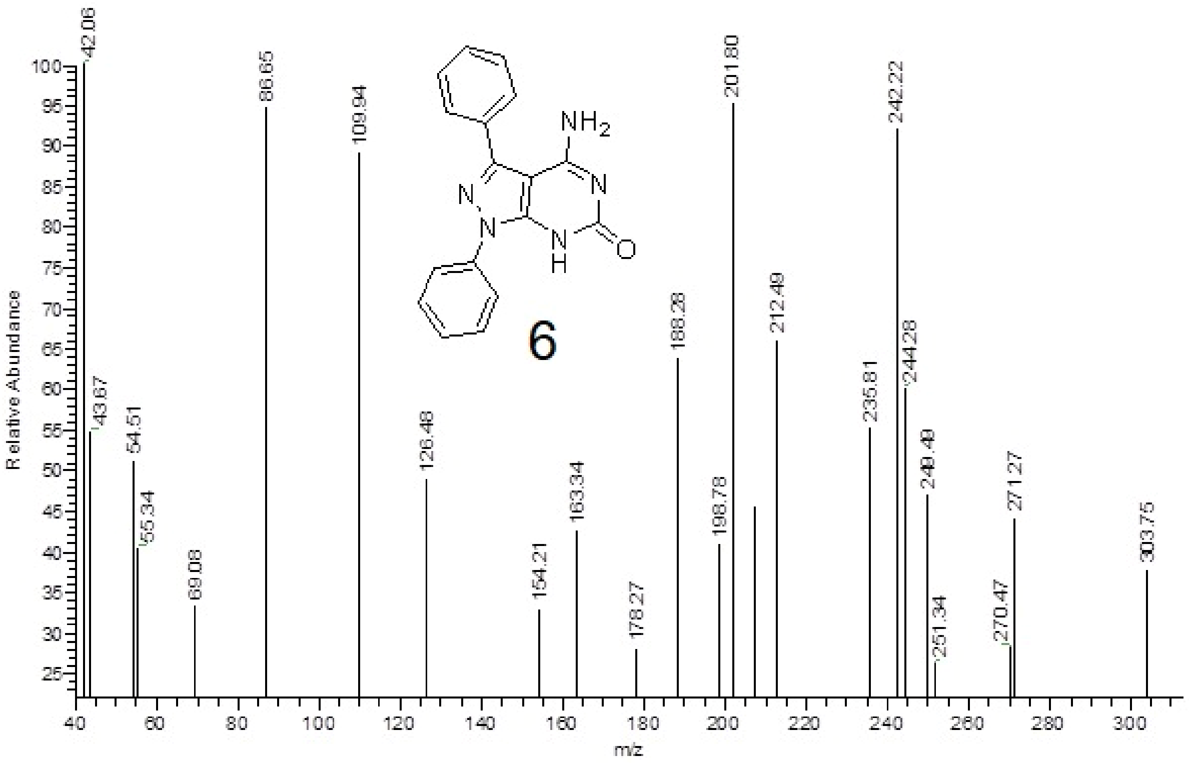

Suppl. Fig. 17 MS of compound 6

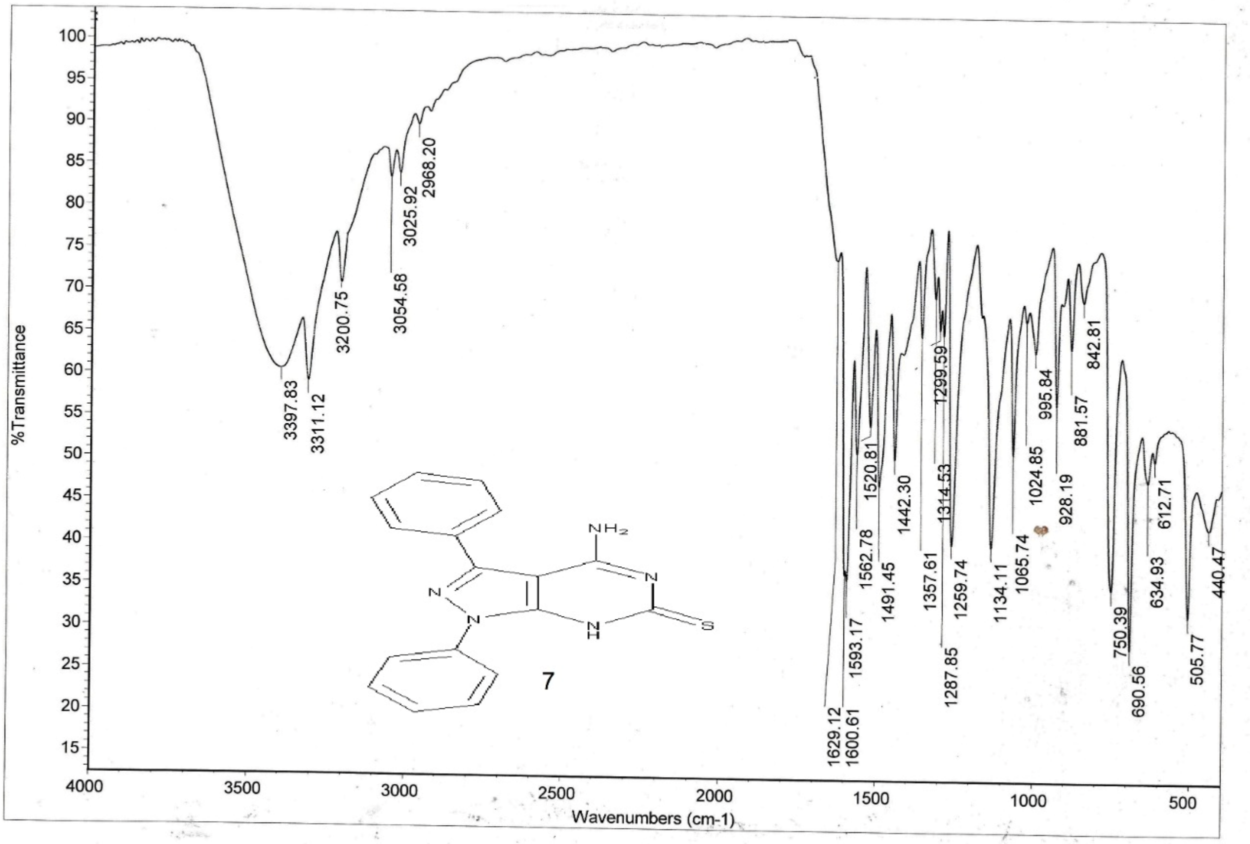

Suppl. Fig. 18 IR spectrum of compound 7

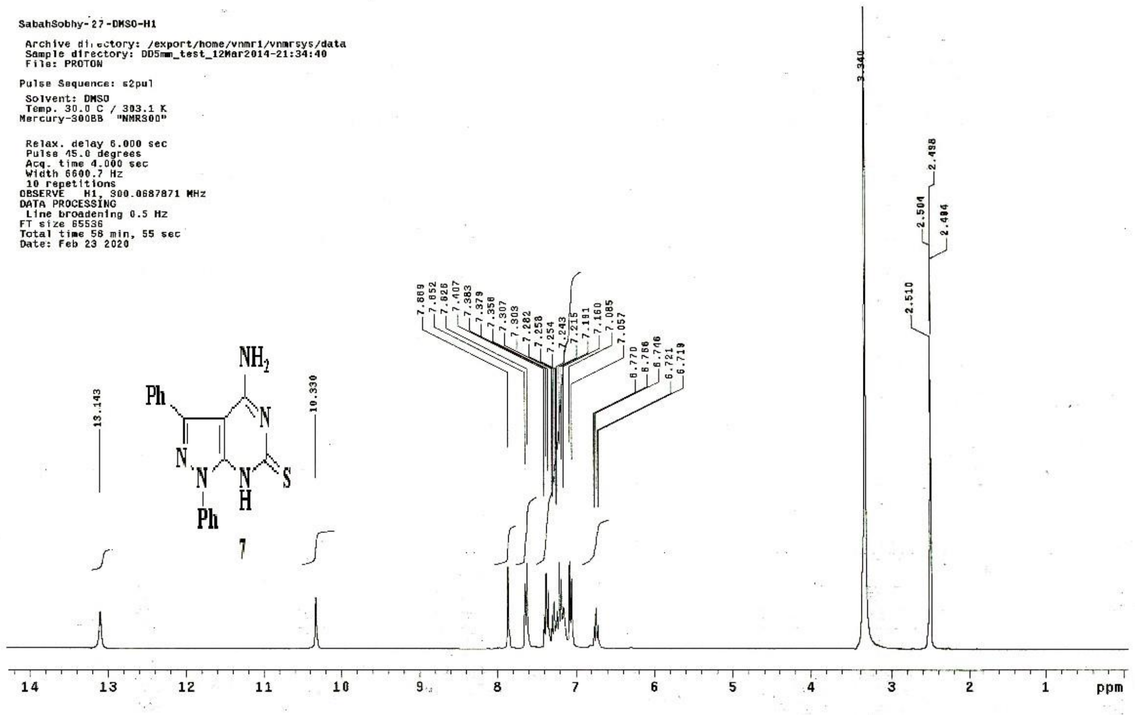

Suppl. Fig. 19 ^{1}H-NMR of compound 7

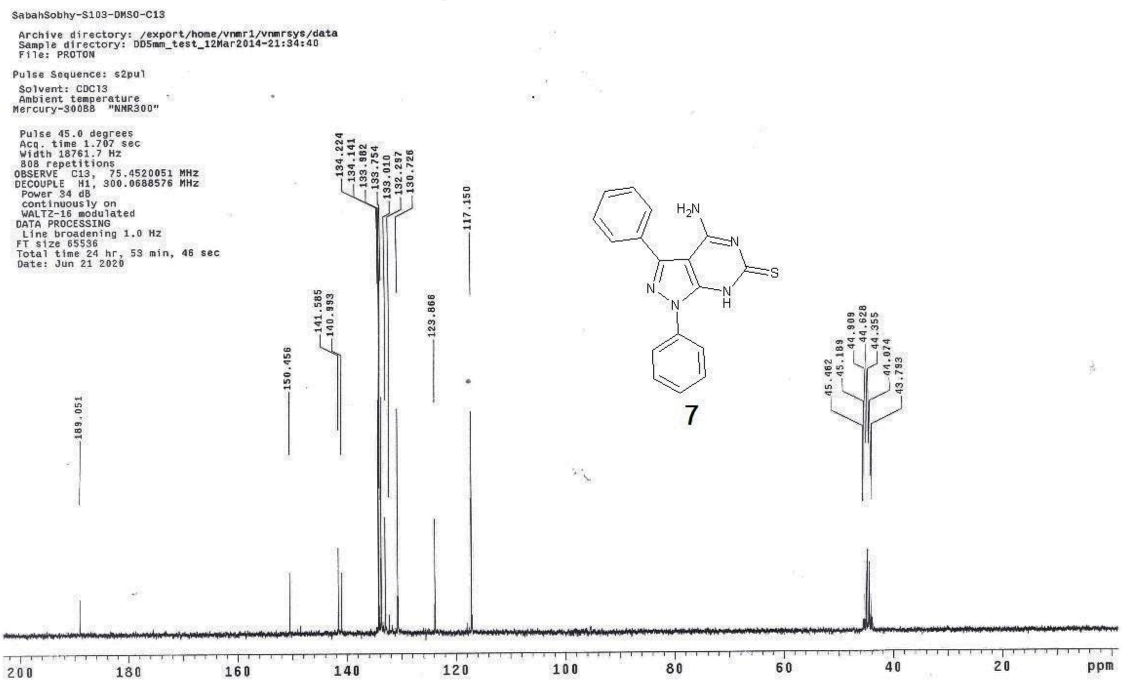

Suppl. Fig. 20 ^{13}C-NMR of compound 7

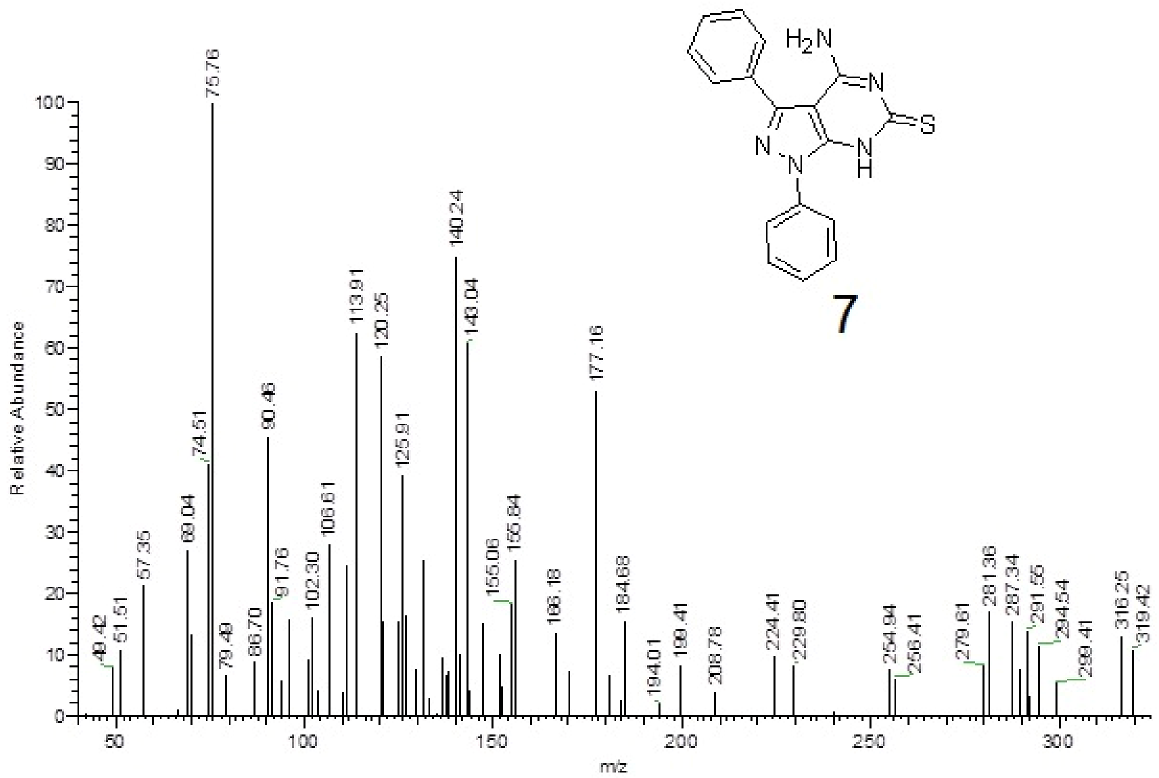

Suppl. Fig. 21 MS of compound 7

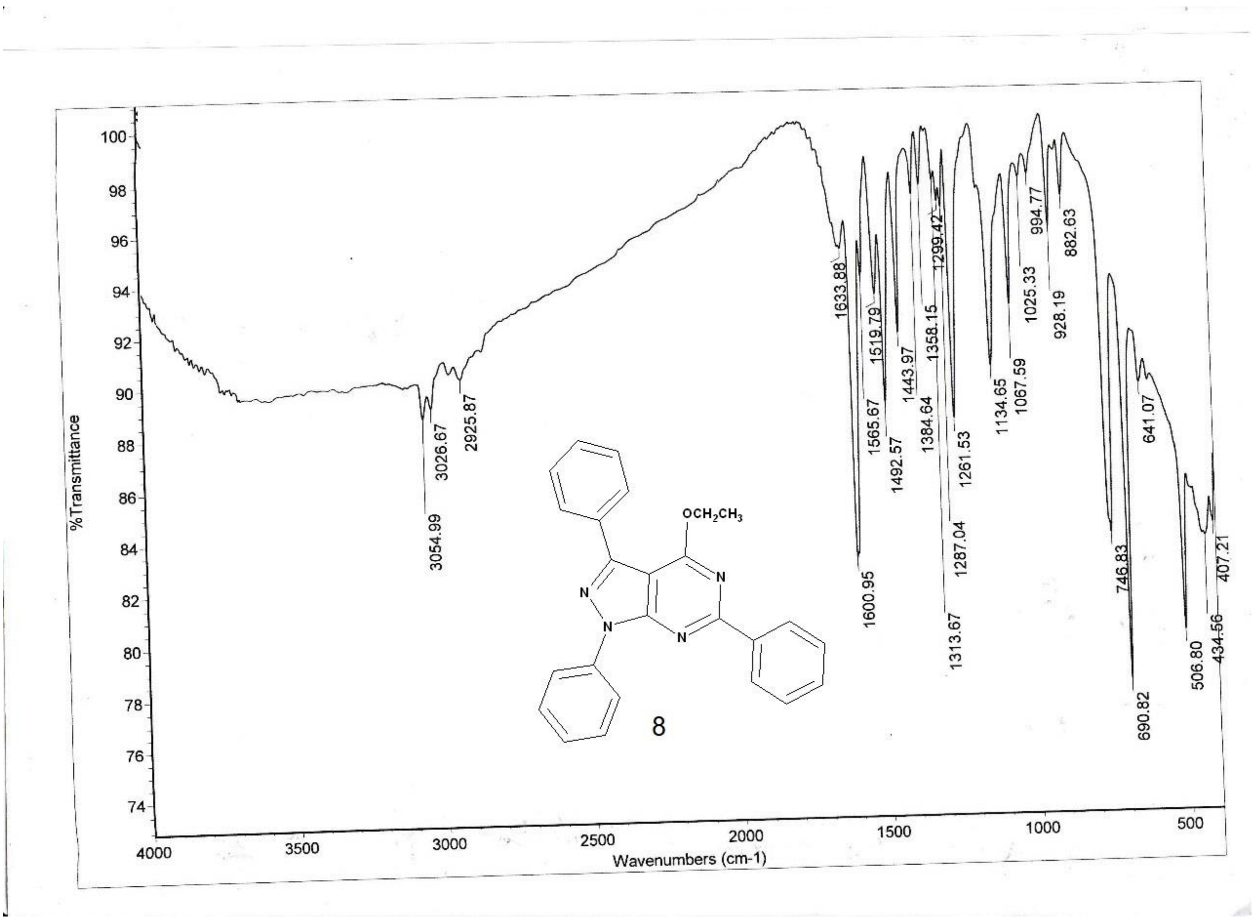

Suppl. Fig. 22 IR spectrum of compound 8

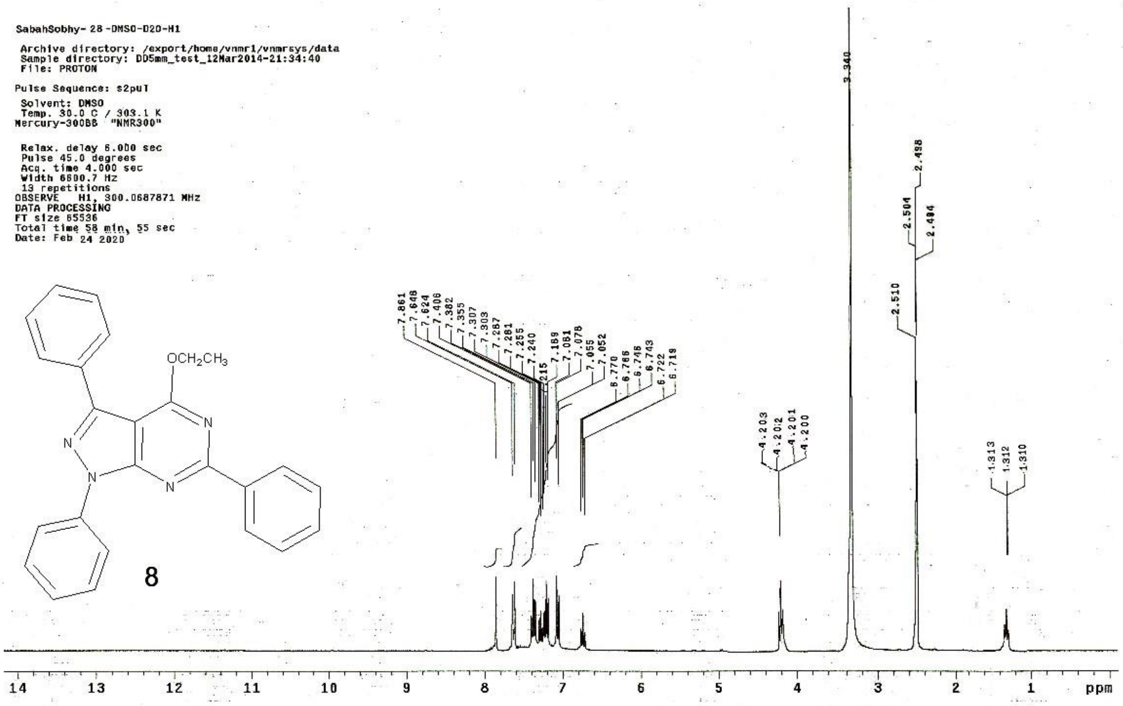

Suppl. Fig. 23 ^{1}H-NMR of compound 8

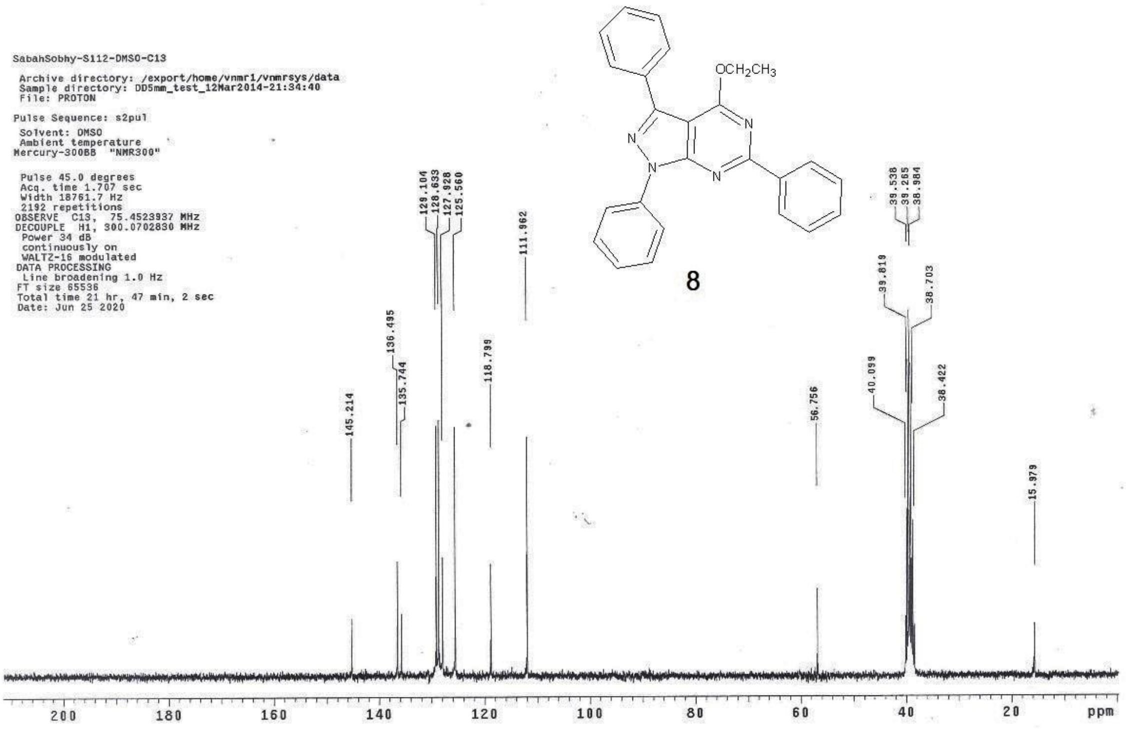

Suppl. Fig. 24 ^{13}C-NMR of compound 8

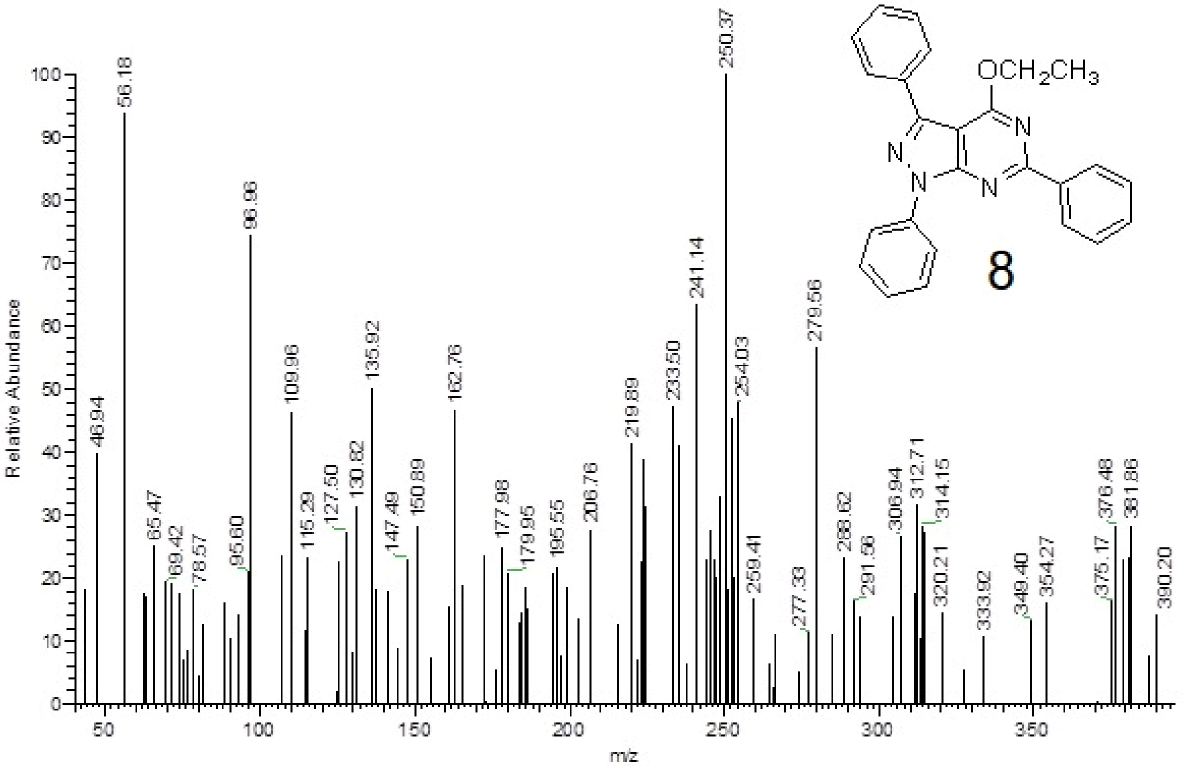

Suppl. Fig. 25 MS of compound 8

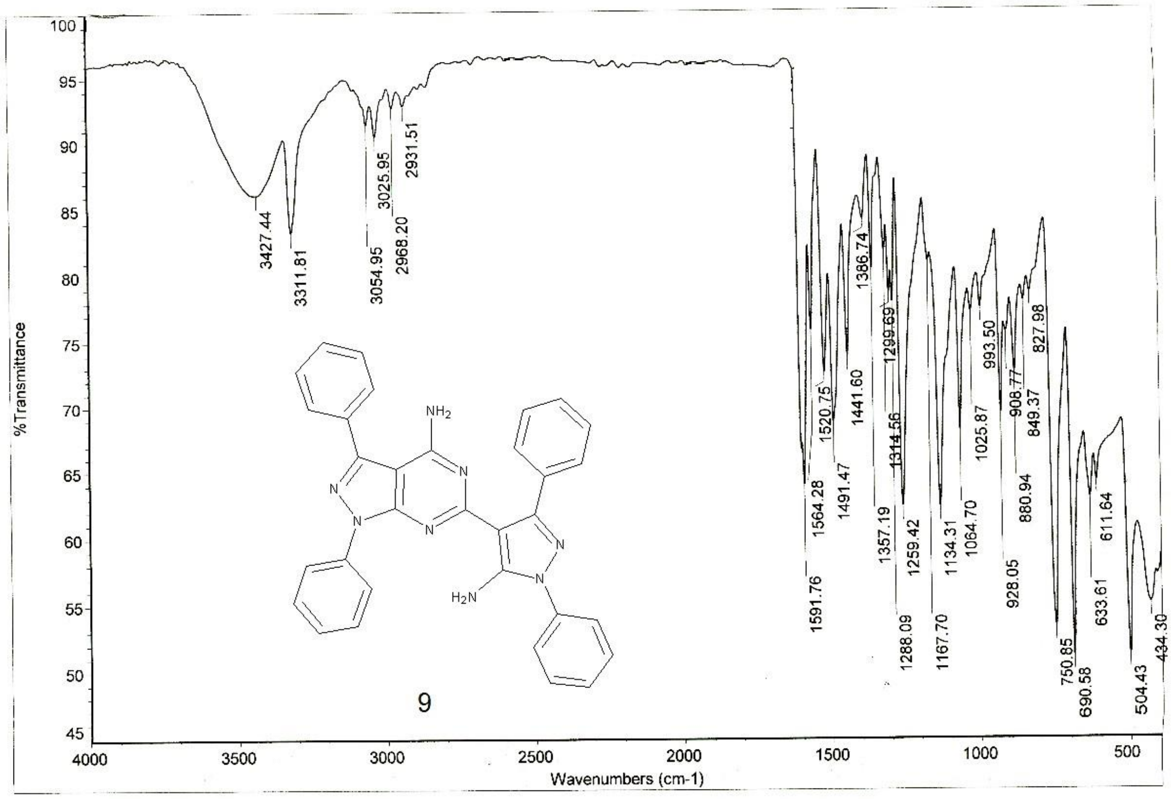

Suppl. Fig. 26 IR spectrum of compound 9

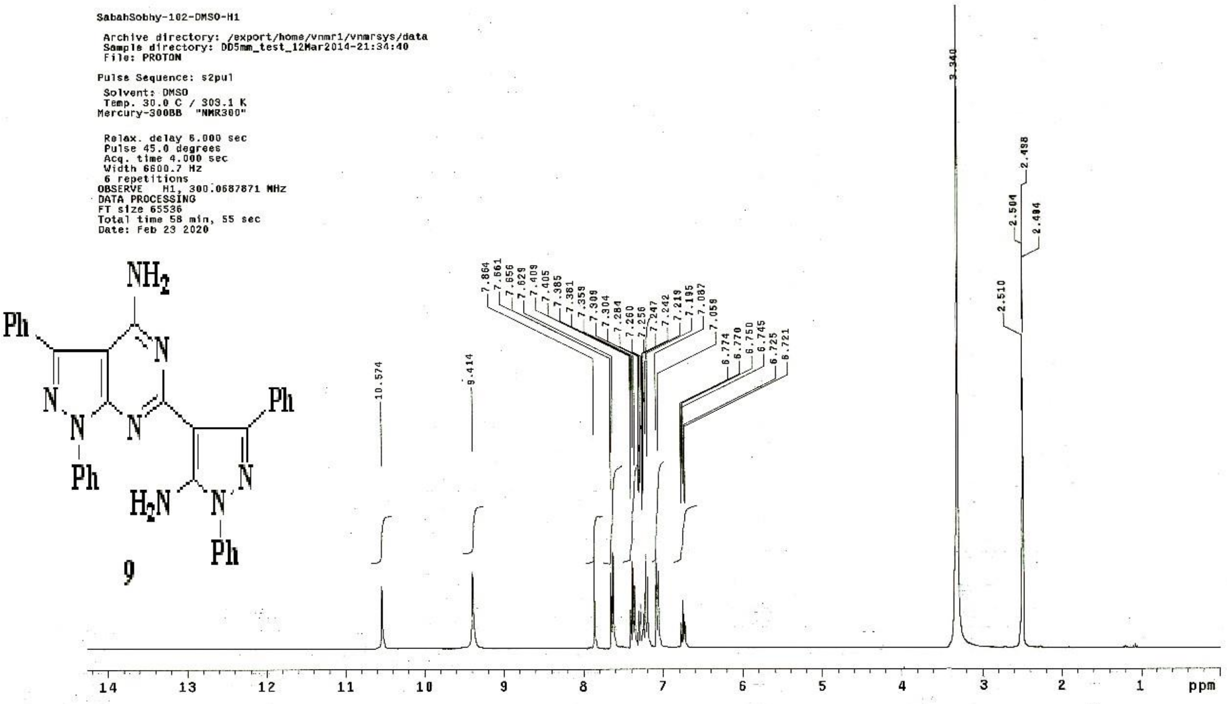

Suppl. Fig. 27 ^{1}H-NMR of compound 9

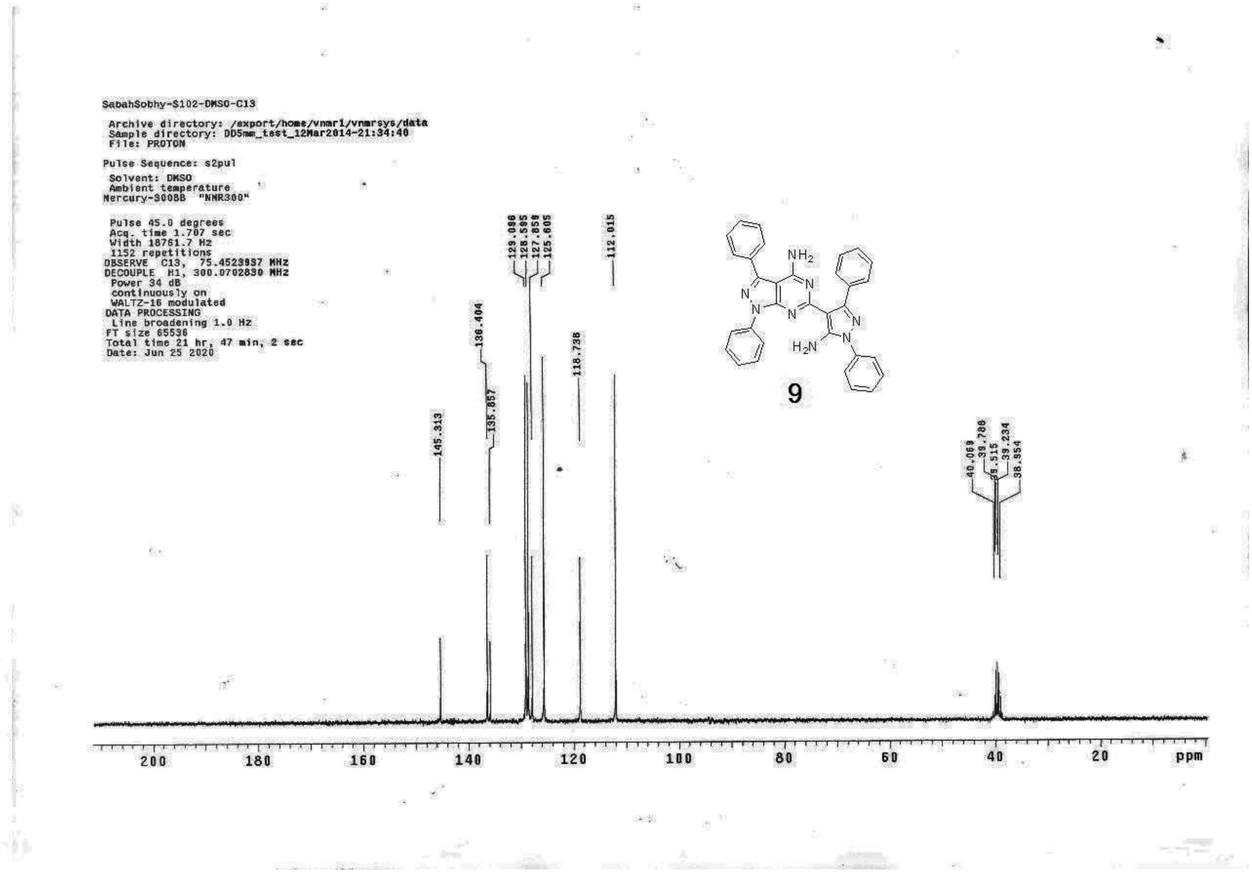

Suppl. Fig. 28 ^{13}C-NMR of compound 9

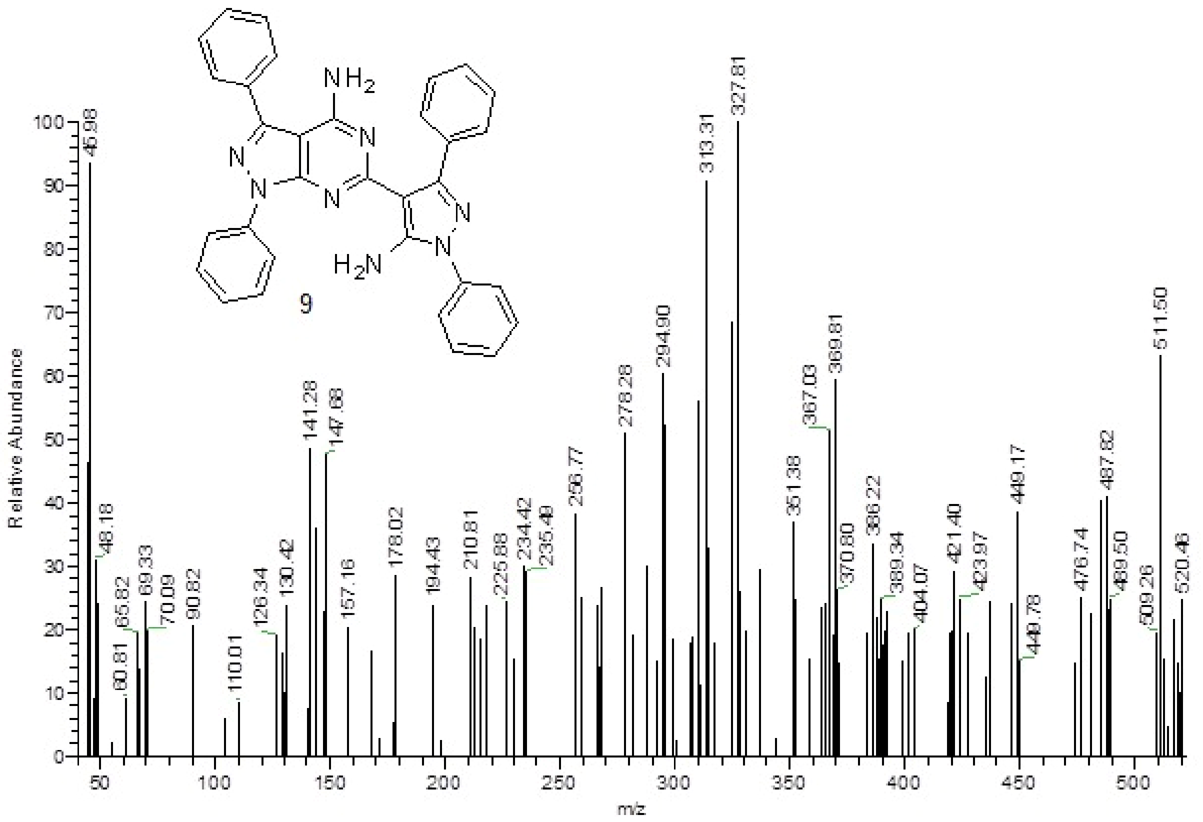

Suppl. Fig. 29 MS of compound 9
